# Supplementary material for: Anti-Adipogenic Activity of Secondary Metabolites Isolated from Smilax sieboldii Miq. on 3T3-L1 Adipocytes
Source: Int J Mol Sci. 2023 May 16;24(10):8866. doi: 10.3390/ijms24108866 (PMC10218641; doi:10.3390/ijms24108866)
Supplement: Supplementary file 1 [file ijms-24-08866-s001.zip › ijms-2406503-supplementary.pdf]

Anti-Adipogenic Activity of Secondary Metabolites Isolated from *Smilax sieboldii* Miq. on 3T3-L1 Adipocytes

Supplementary Material

List of Figures

Figure S1. Effect of crude extract of *S. sieboldii* whole plant on cell viability by using MTT

Figure S2. ^{1}H-NMR spectrum (acetone-d_6, 700 MHz) of compound **1**

Figure S3. ^{13}C-NMR spectrum (acetone-d_6, 175 MHz) of the compound **1**

Figure S4. ^{1}H-NMR spectrum (acetone-d_6, 700 MHz) of the compound **2**

Figure S5. ^{13}C-NMR spectrum (acetone-d_6, 175 MHz) of the compound **2**

Figure S6. ^{1}H-NMR spectrum (CD_3OD, 700 MHz) of the compound **3**

Figure S7. ^{13}C-NMR spectrum (CD_3OD, 175 MHz) of the compound **3**

Figure S8. ^{1}H-NMR spectrum (CD_3OD, 700 MHz) of the compound **4**

Figure S9. ^{13}C-NMR spectrum (CD_3OD, 175 MHz) of the compound **4**

Figure S10. ^{1}H-NMR spectrum (CD_3OD, 700 MHz) of the compound **5**

Figure S11. ^{13}C-NMR spectrum (CD_3OD, 175 MHz) of the compound **5**

Figure S12. ^{1}H-NMR spectrum (CD_3OD, 700 MHz) of the compound **6**

Figure S13. ^{13}C-NMR spectrum (CD_3OD, 175 MHz) of the compound **6**

Figure S14. ^{1}H-NMR spectrum (CD_3OD, 700 MHz) of the compound **7**

Figure S15. ^{13}C-NMR spectrum (CD_3OD, 175 MHz) of the compound **7**

Figure S16. ^{1}H-NMR spectrum (CD_3OD, 700 MHz) of the compound **8**

Figure S17. ^{13}C-NMR spectrum (CD_3OD, 175 MHz) of the compound **8**

Figure S18. ^{1}H-NMR spectrum (CD_3OD, 700 MHz) of the compound **9**

Figure S19. ^{13}C-NMR spectrum (CD_3OD, 175 MHz) of the compound **9**

Figure S20. ^{1}H-NMR spectrum (CD_3OD, 700 MHz) of the compound **10**

Figure S21. ^{13}C-NMR spectrum (CD_3OD, 175 MHz) of the compound **10**

Figure S22. ^{1}H-NMR spectrum (CD_3OD, 700 MHz) of the compound **11**

Figure S23. ^{13}C-NMR spectrum (CD_3OD, 175 MHz) of the compound **11**

Figure S24. ^{1}H-NMR spectrum (CD_3OD, 700 MHz) of the compound **12**

Figure S25. ^{13}C-NMR spectrum (CD_3OD, 175 MHz) of the compound **12**

Figure S26. ^{1}H-NMR spectrum (CD_3OD, 700 MHz) of the compound **13**

Figure S27. ^{13}C-NMR spectrum (CD_3OD, 175 MHz) of the compound **13**

Figure S28. ^{1}H-NMR spectrum (CD_3OD, 700 MHz) of the compound **14**

Figure S29. ^{13}C-NMR spectrum (CD_3OD, 175 MHz) of the compound **14**

Figure S30. ^{1}H-NMR spectrum (CD_3OD, 700 MHz) of the compound **15**

Figure S31. ^{13}C-NMR spectrum (CD_3OD, 175 MHz) of the compound **15**

Figure S32. ^{1}H-NMR spectrum (CD_3OD, 700 MHz) of the compound **16**

Figure S33. ^{13}C-NMR spectrum (CD_3OD, 175 MHz) of the compound **16**

Figure S34. DEPT135 spectrum of the compound **16**

Figure S35. ^{1}H-^{1}H COSY spectrum of the compound **16**

Figure S36. HSQC spectrum of the compound **16**

Figure S37. HMBC spectrum of the compound **16**

Figure S38. Expansion of HMBC spectrum of compound **16**

Figure S39. HRESIMS spectrum of the compound **16**

Figure S40. ^{1}H-NMR spectrum ($CDCl_3$, 700 MHz) of the compound **17**

Figure S41. ^{13}C-NMR spectrum ($CDCl_3$, 175 MHz) of the compound **17**

Figure S42. DEPT135 spectrum of the compound **17**

Figure S43. ^{1}H-^{1}H COSY spectrum of the compound **17**

Figure S44. HSQC spectrum of the compound **17**

Figure S45. HMBC spectrum of the compound **17**

Figure S46. ROESY spectrum of compound **17**

Figure S47. Specific optical rotation of compound **17**

Figure S48. IR spectrum of compound **17**

Figure S49. HRESIMS spectrum of the compound **17**

Figure S50. ^{1}H-NMR spectrum ($CDCl_3$, 700 MHz) of the compound **18**

Figure S51. ^{13}C-NMR spectrum ($CDCl_3$, 175 MHz) of the compound **18**

Figure S52. DEPT135 spectrum of the compound **18**

Figure S53. ^{1}H-^{1}H COSY spectrum of the compound **18**

Figure S54. HSQC spectrum of the compound **18**

Figure S55. HMBC spectrum of the compound **18**

Figure S56. ROESY spectrum of compound **18**

Figure S57. Specific optical rotation of compound **18**

Figure S58. IR spectrum of compound **18**

Figure S59. HRESIMS spectrum of the compound **18**

Figure S60. ^{1}H-NMR spectrum (CD_3OD, 700 MHz) of the compound **19**

Figure S61. ^{13}C-NMR spectrum (CD_3OD, 175 MHz) of the compound **19**

1. General Experimental Procedure

Optical rotations were measured using a JASCO P-2000 polarimeter (Tokyo, Japan). UV and FT-IR spectra were measured on a JASCO UV-550 and JASCO Model FT/IR-4100 spectrometer (Tokyo, Japan), respectively. Nuclear magnetic resonance (NMR) spectra were recorded on a Bruker Ascend III 700 spectrometer (Bruker BioSpin GmbH., Rheinstetten, Germany) in acetone-d_6, $CDCl_3$, and CD_3OD at room temperature. Chemical shifts are in ppm (δ), relative to tetramethylsilane as an internal standard, and coupling constants are in hertz; ^1H, ^{13}C, DEPT, COSY, HSQC, and HMBC by using the standard pulse sequences. Electrospray ionization mass (ESI-MS) spectra were acquired on an Agilent 6130 series quadrupole LC/MS System (Agilent Technologies, CA, USA). HRESIMS spectra were recorded by a Triple TOF 5600+ mass spectrometer (AB SCIEX, MA, USA). Open column chromatography was performed using Diaion HP-20 adsorbent resin (Mitsubishi Chemical Corp, Tokyo, Japan). Medium-pressure liquid chromatography (MPLC) was conducted using a CombiFlash Rf flash chromatography system (Teledyne ISCO Inc., NE, USA), and the separations were performed on a Redi*Sep*® Rf C_{18} column with a flow rate of 40 mL/min. Preparative high-performance liquid chromatography (HPLC) was performed on a Thermo Scientifc Dionex Ultimate 3000 UHPLC system (Thermo Fisher Scientific Inc., MA, USA) equipped with an HPG-3200BX biocompatible binary semipreparative pump and a rapid separations PDA detector (Ultimate DAD-3000) controlled by Chromeleon 7.2 software. The separations were carried out on a Kromasil 100-5-C18 column (5 μm, 21.2 × 250 mm, Nouryon Chemicals Finance B.V., Amsterdam, Netherlands). Thin layer chromatography (TLC) was performed using DC-Fertigfolien ALUGRAM® SIL G/$UV_{254+366}$ (0.2 mm, Macherey-Nagel GmbH & Co. KG, Düren, Germany) plates, and spots were visualized by a 10% vanillin−sulfuric acid reagent. All chemicals and solvents were of analytical grade and were used without further purification.

Table S1. *In vitro* cytotoxicity of compounds **1**–**19**.

Compounds	**1**	**2**	**3**	**4**	**5**	**6**	**7**	**8**	**9**	**10**	**11**	**12**	**13**	**14**	**15**	**16**	**17**	**18**	**19**
IC_{50} (μM)	NC	NC	NC	NC	NC	NC	NC	NC	NC	NC	NC	NC	NC	NC	NC	NC	NC	NC	NC

IC_{50} values of tested compounds were measured. 3T3-L1 preadipocytes (40% confluence) were treated with different concentrations (25, 50, and 100 μM) of tested compounds for 48 h. Statistical analysis was performed by Student's *t*-test.
NC, no cytotoxicity.

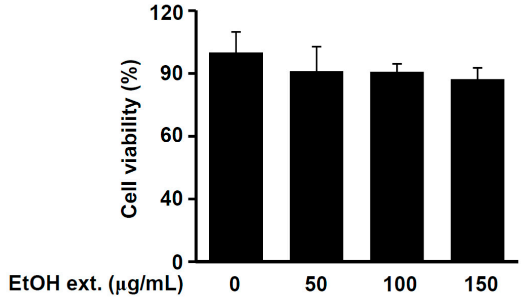

Figure S1. Effect of crude extract of *S. sieboldii* whole plant on cell viability by using MTT

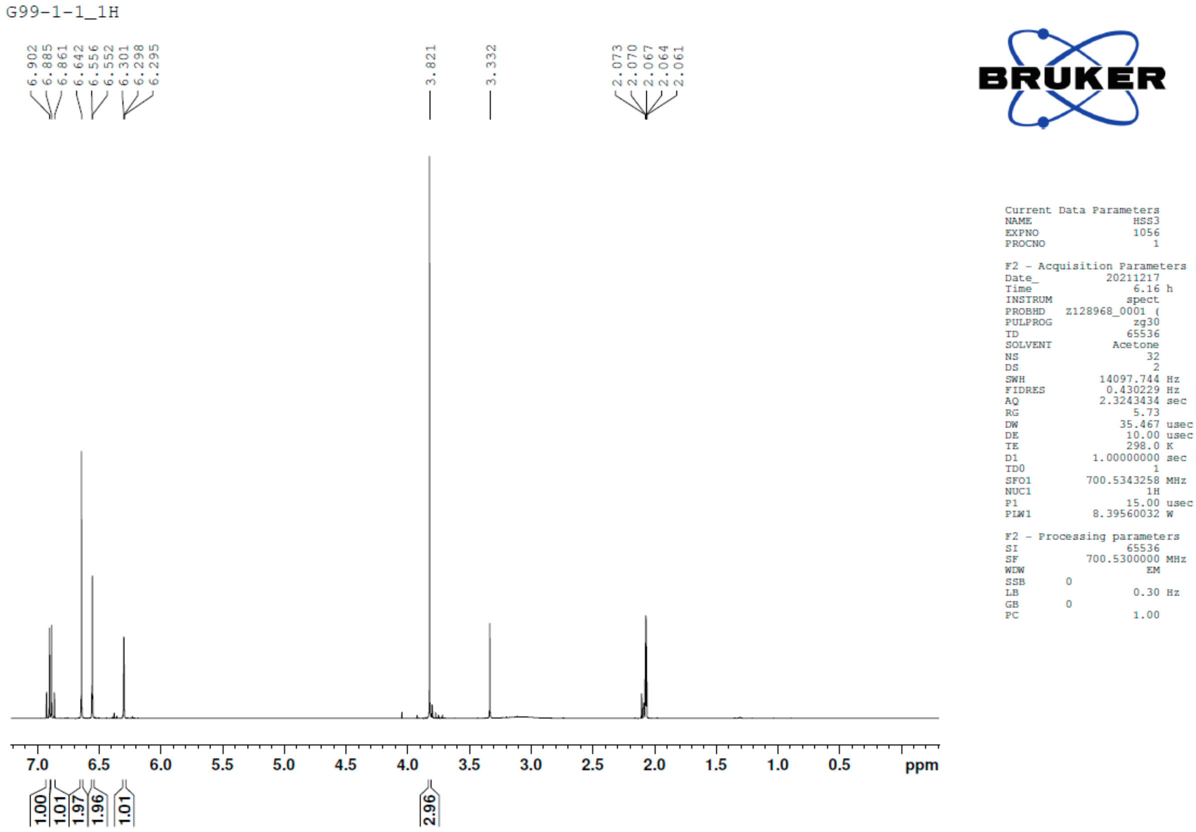

Figure S2. ^{1}H-NMR spectrum (acetone-d_6, 700 MHz) of compound **1**

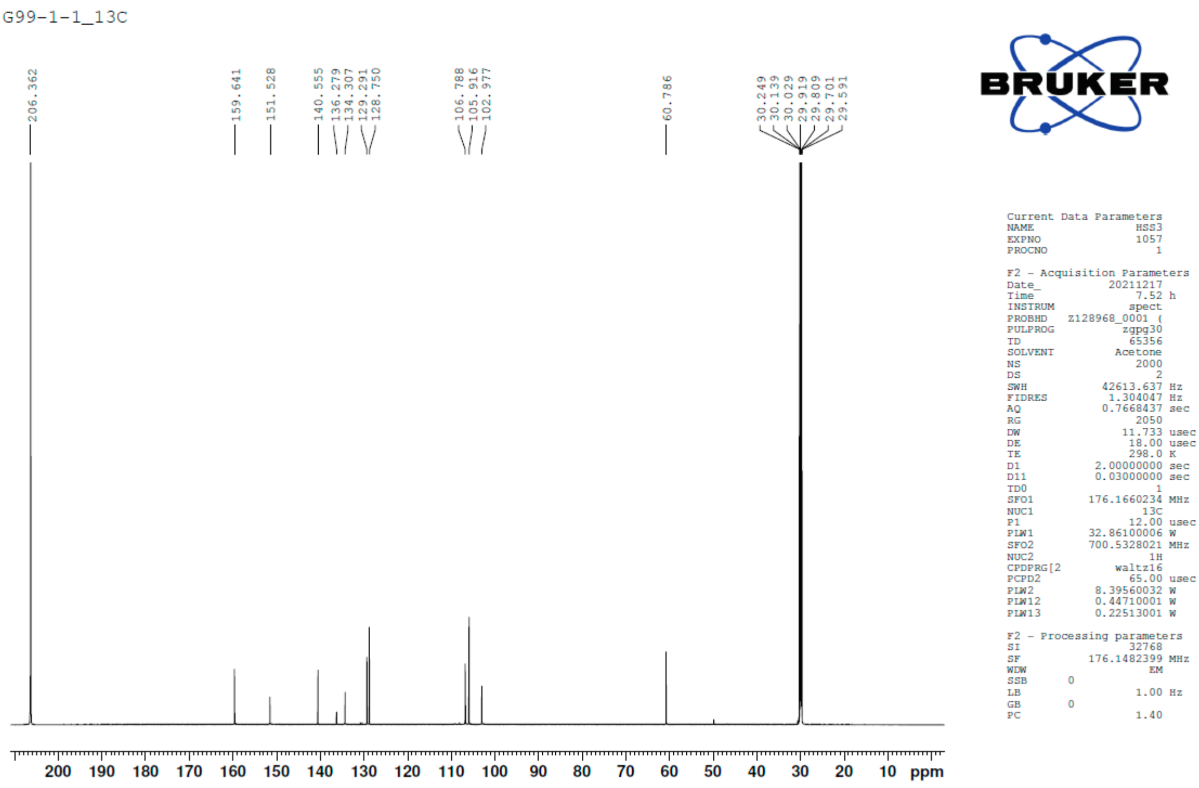

Figure S3. ^{13}C-NMR spectrum (acetone-d_6, 175 MHz) of compound **1**

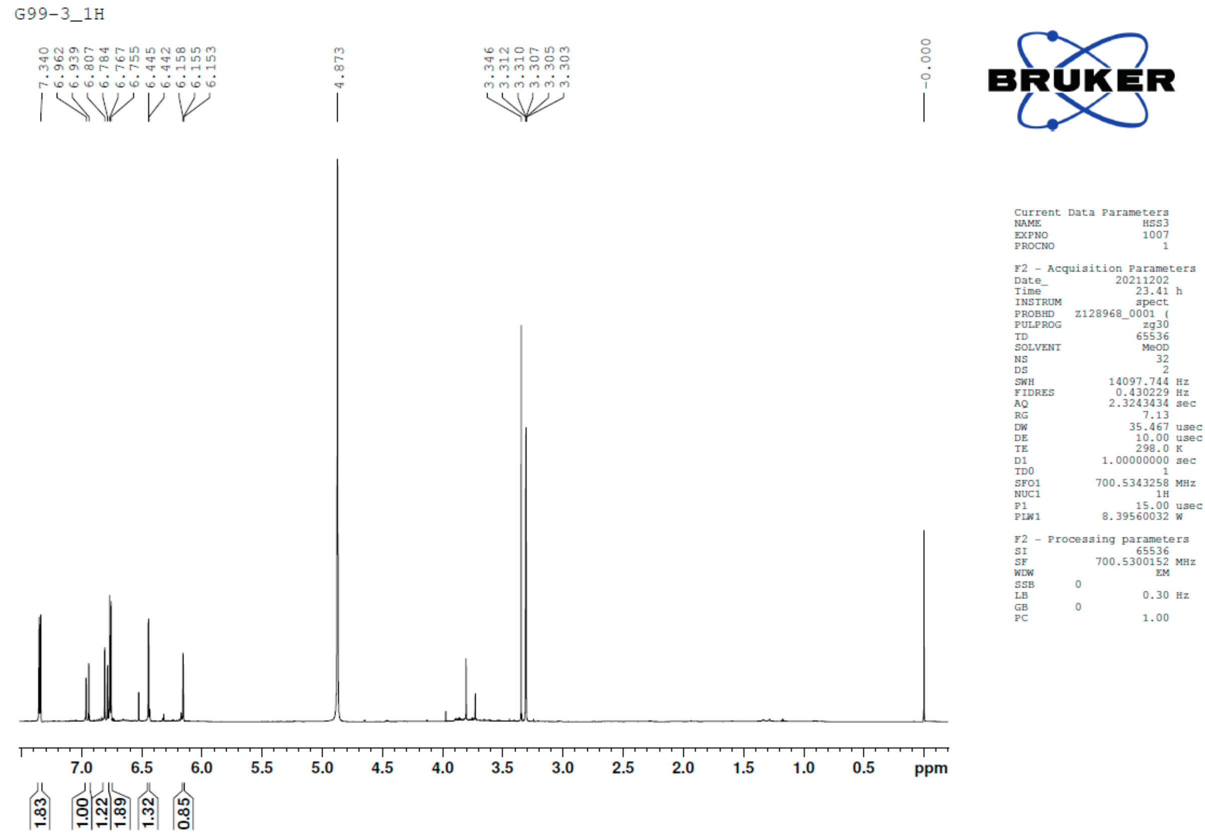

Figure S4. ^{1}H-NMR spectrum (acetone-d_6, 700 MHz) of compound **2**

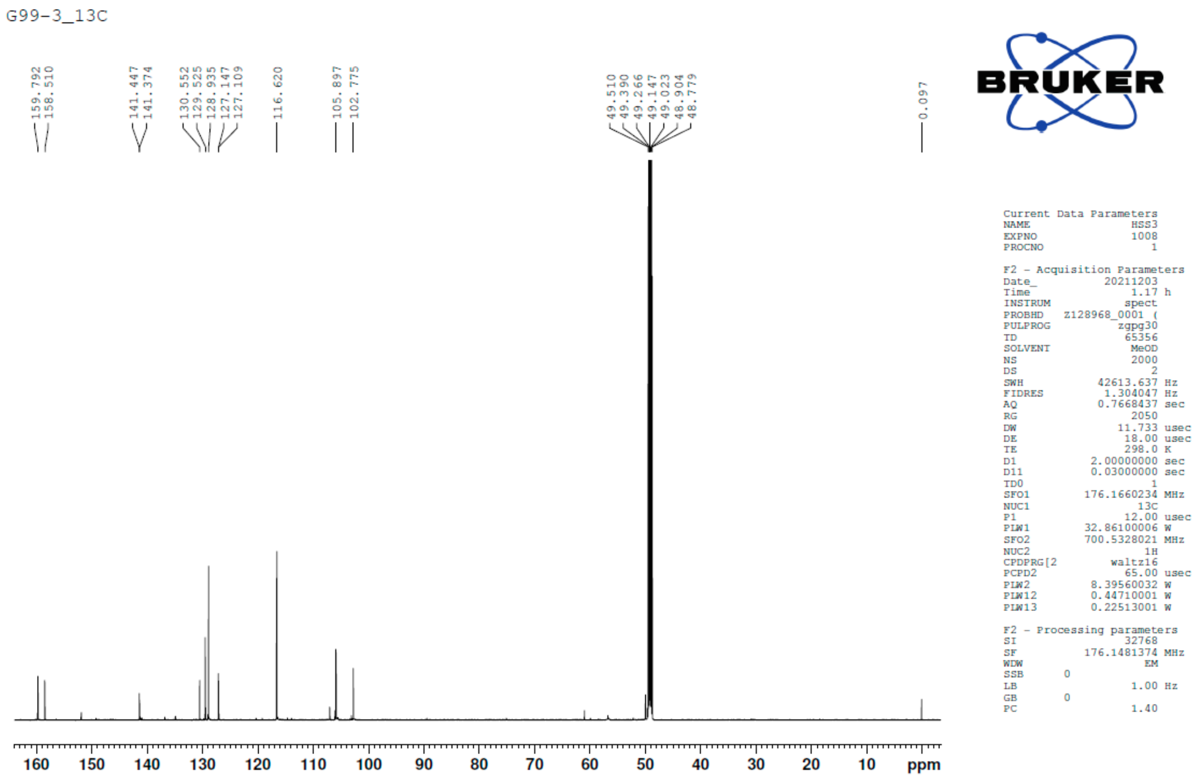

Figure S5. ^{13}C-NMR spectrum (acetone-d_6, 175 MHz) of compound **2**

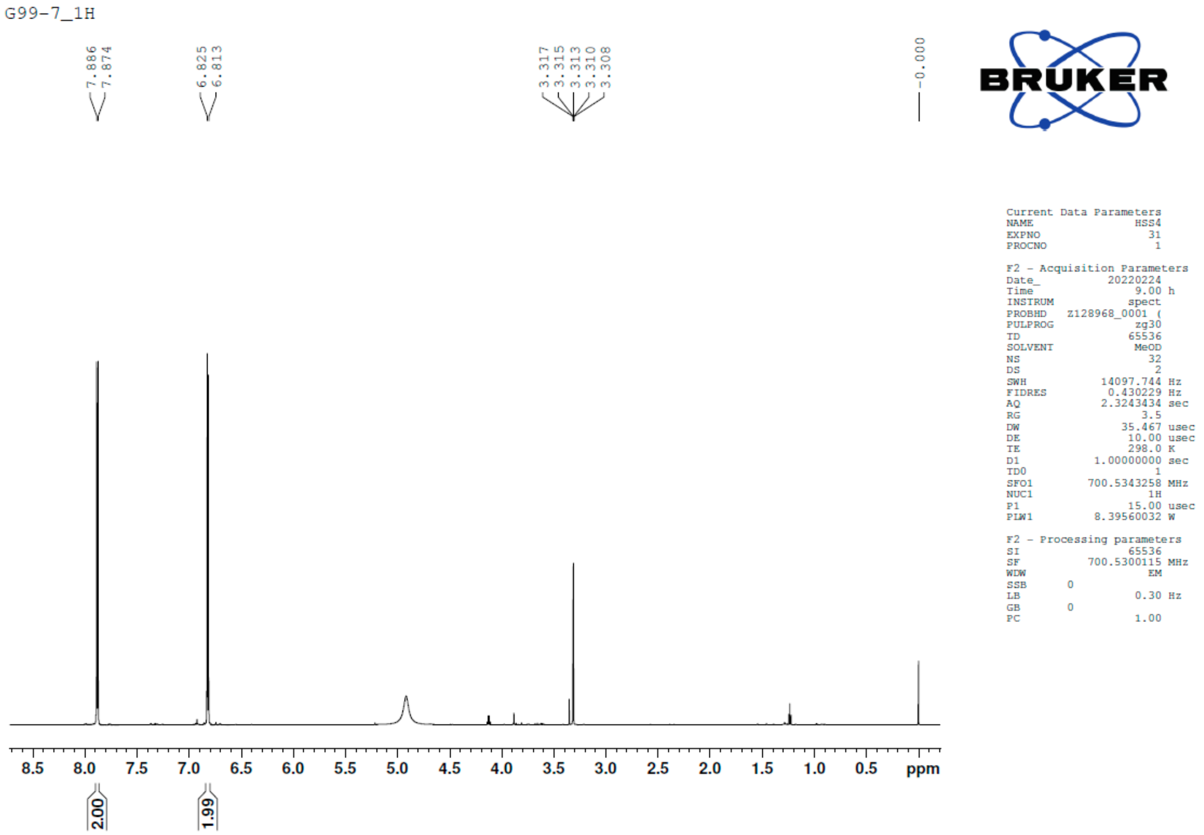

Figure S6. ^{1}H-NMR spectrum (CD_3OD, 700 MHz) of compound **3**

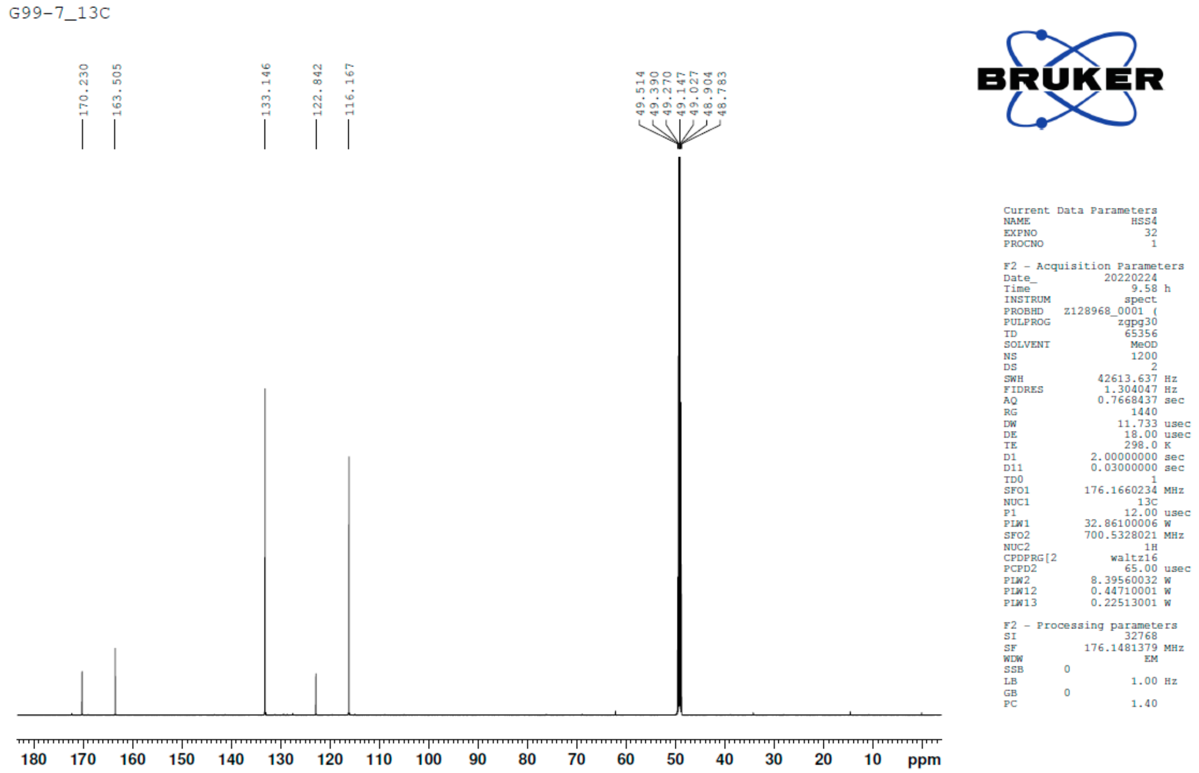

Figure S7. ^{13}C-NMR spectrum (CD$_3$OD, 175 MHz) of compound **3**

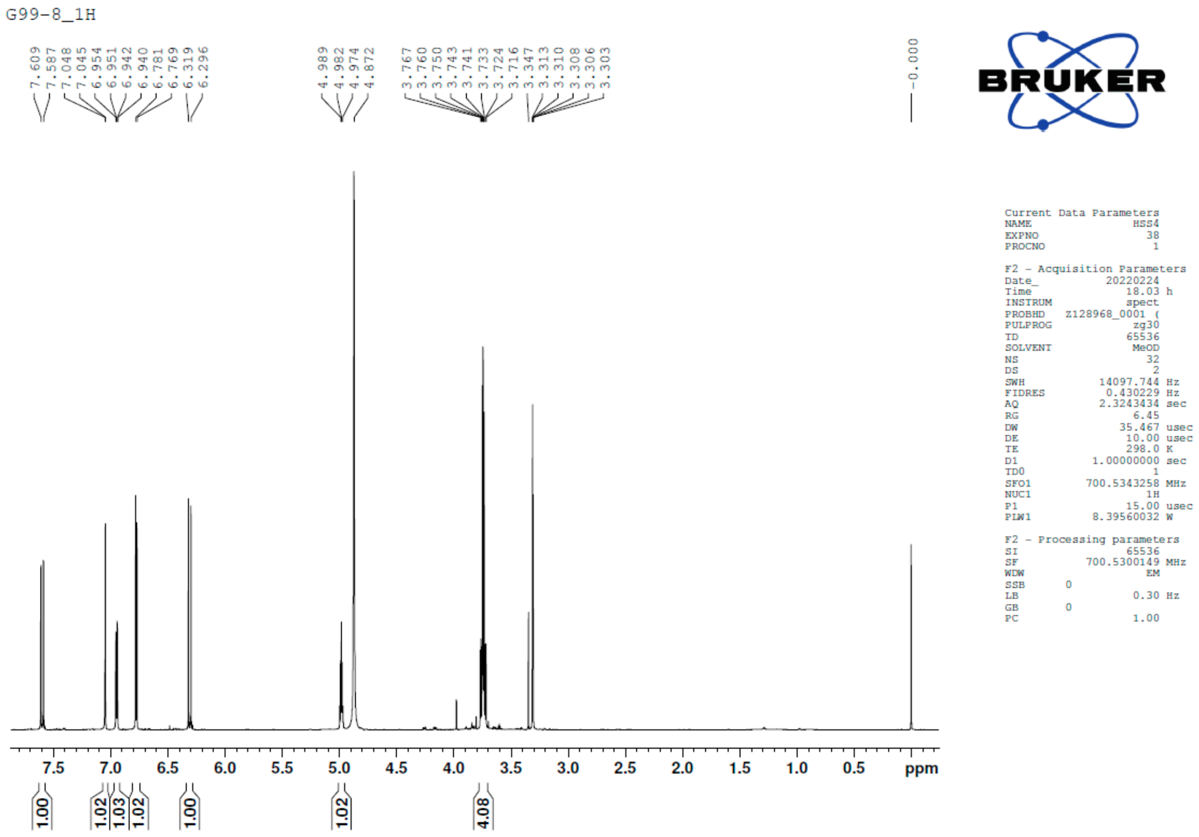

Figure S8. ^{1}H-NMR spectrum (CD$_3$OD, 700 MHz) of compound **4**

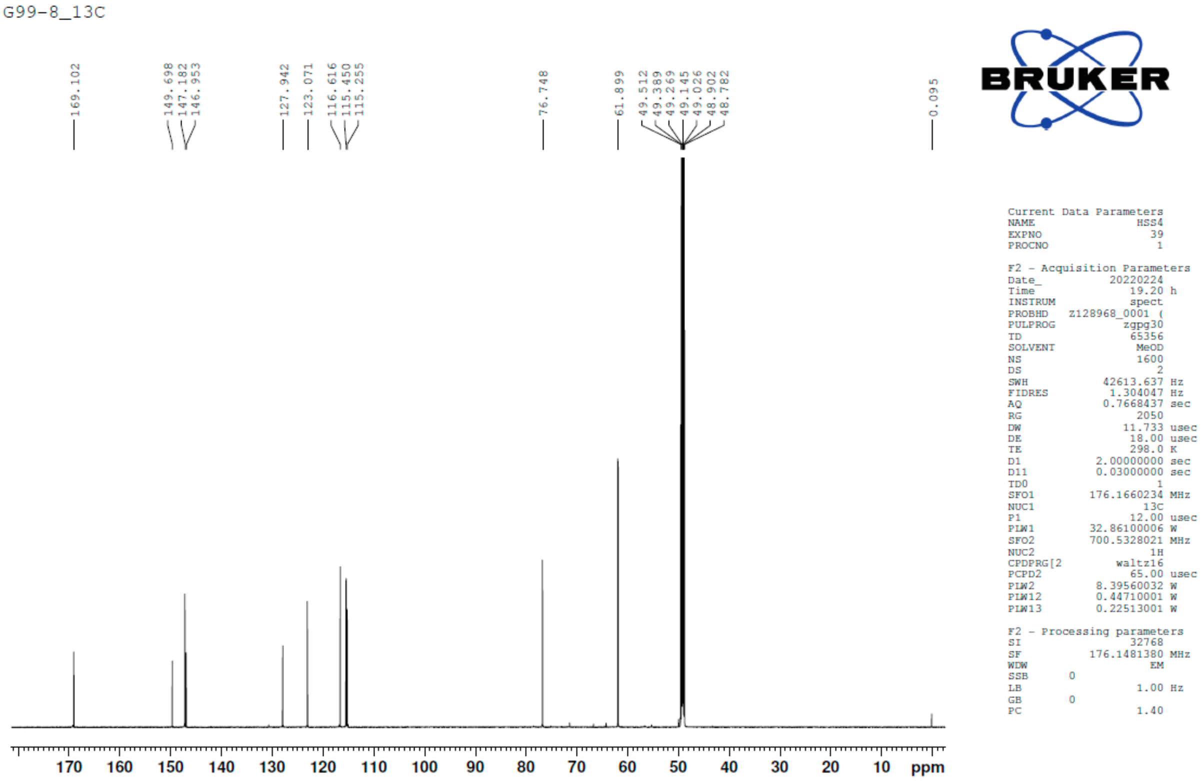

Figure S9. ^{13}C-NMR spectrum (CD_3OD, 175 MHz) of compound **4**

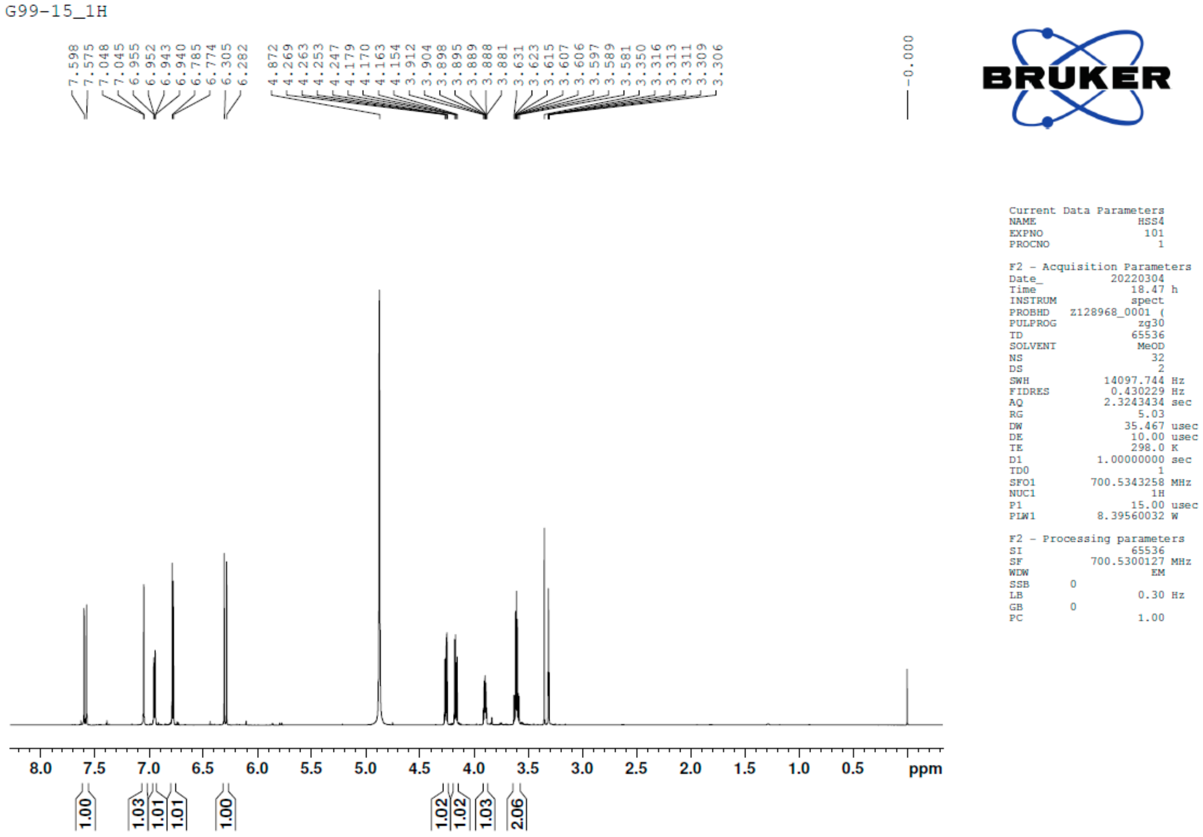

Figure S10. ^{1}H-NMR spectrum (CD_3OD, 700 MHz) of compound **5**

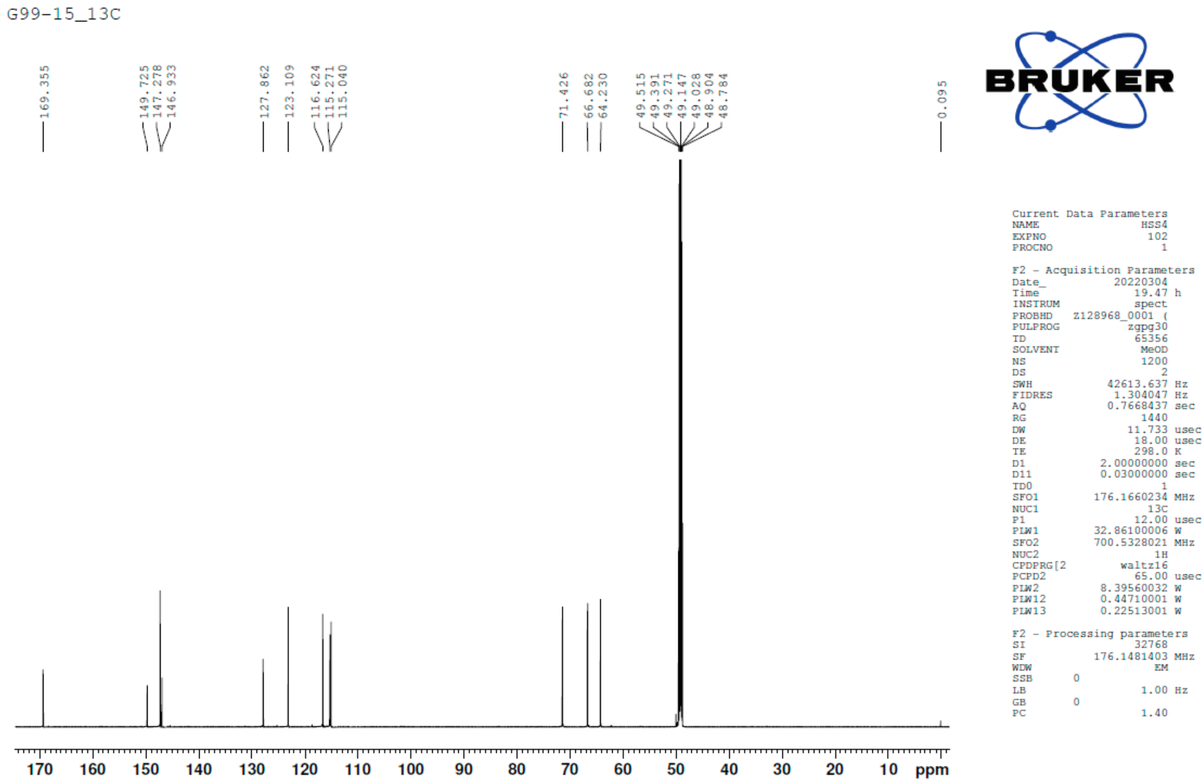

Figure S11. ^{13}C-NMR spectrum (CD_3OD, 175 MHz) of compound **5**

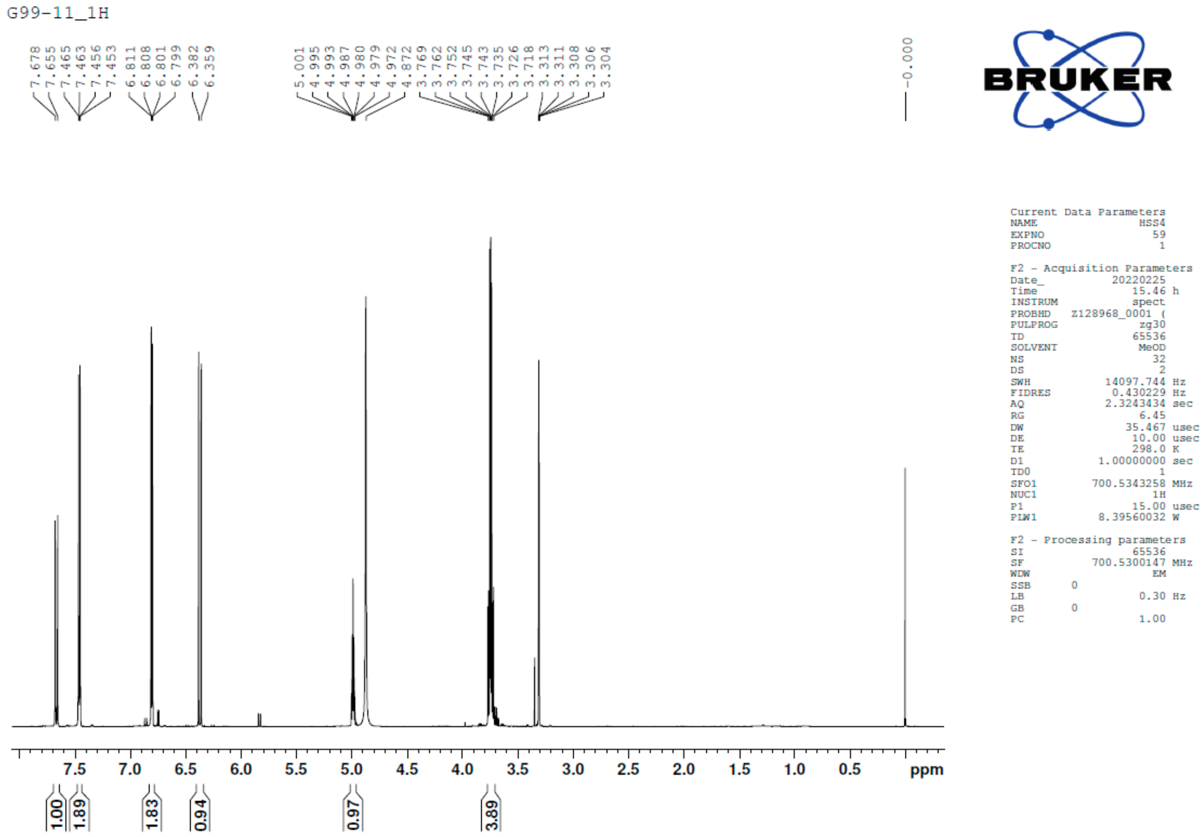

Figure S12. ^{1}H-NMR spectrum (CD_3OD, 700 MHz) of compound **6**

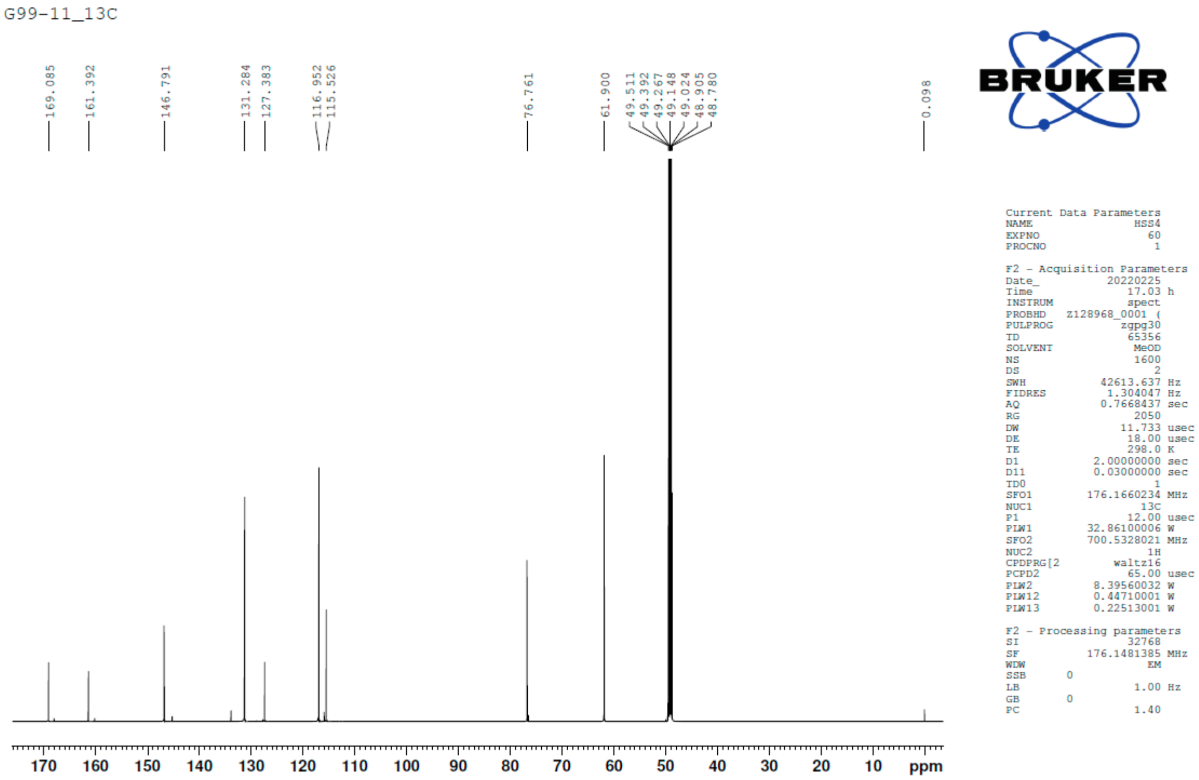

Figure S13. ^{13}C-NMR spectrum (CD_3OD, 175 MHz) of compound **6**

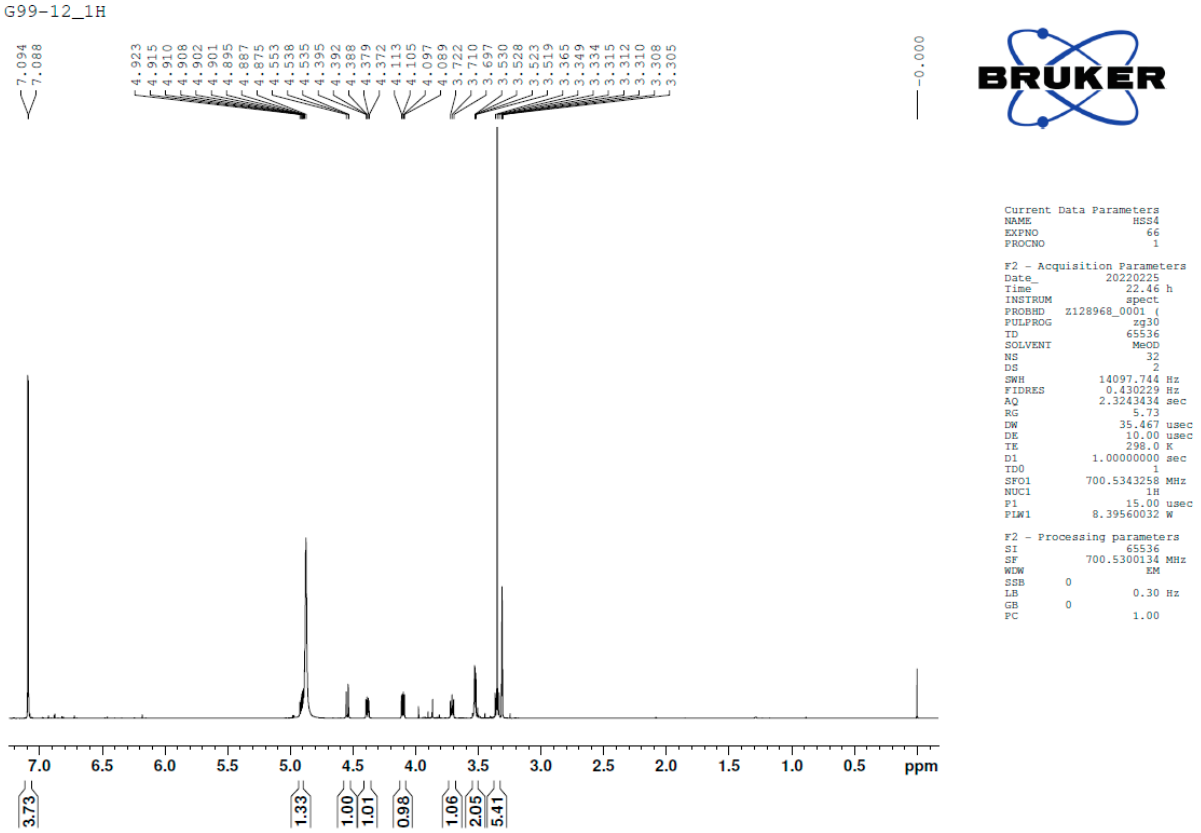

Figure S14. ^{1}H-NMR spectrum (CD_3OD, 700 MHz) of compound **7**

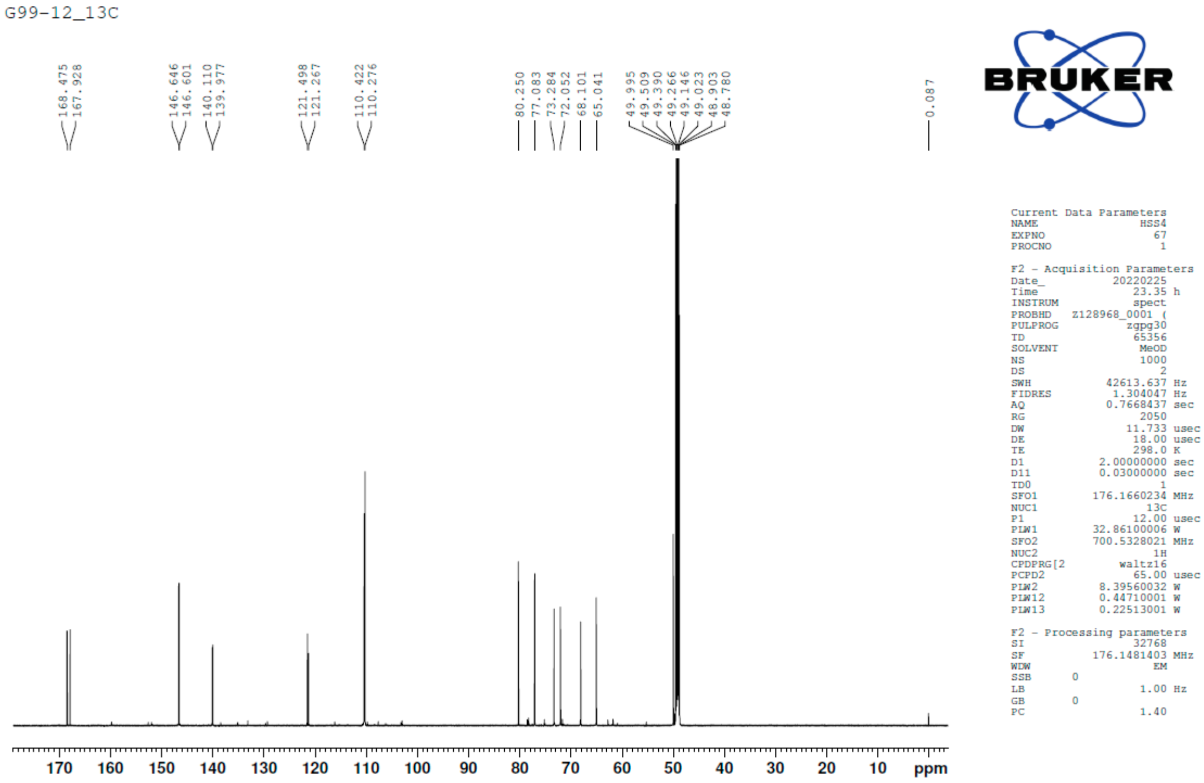

Figure S15. ^{13}C-NMR spectrum (CD_3OD, 175 MHz) of compound **7**

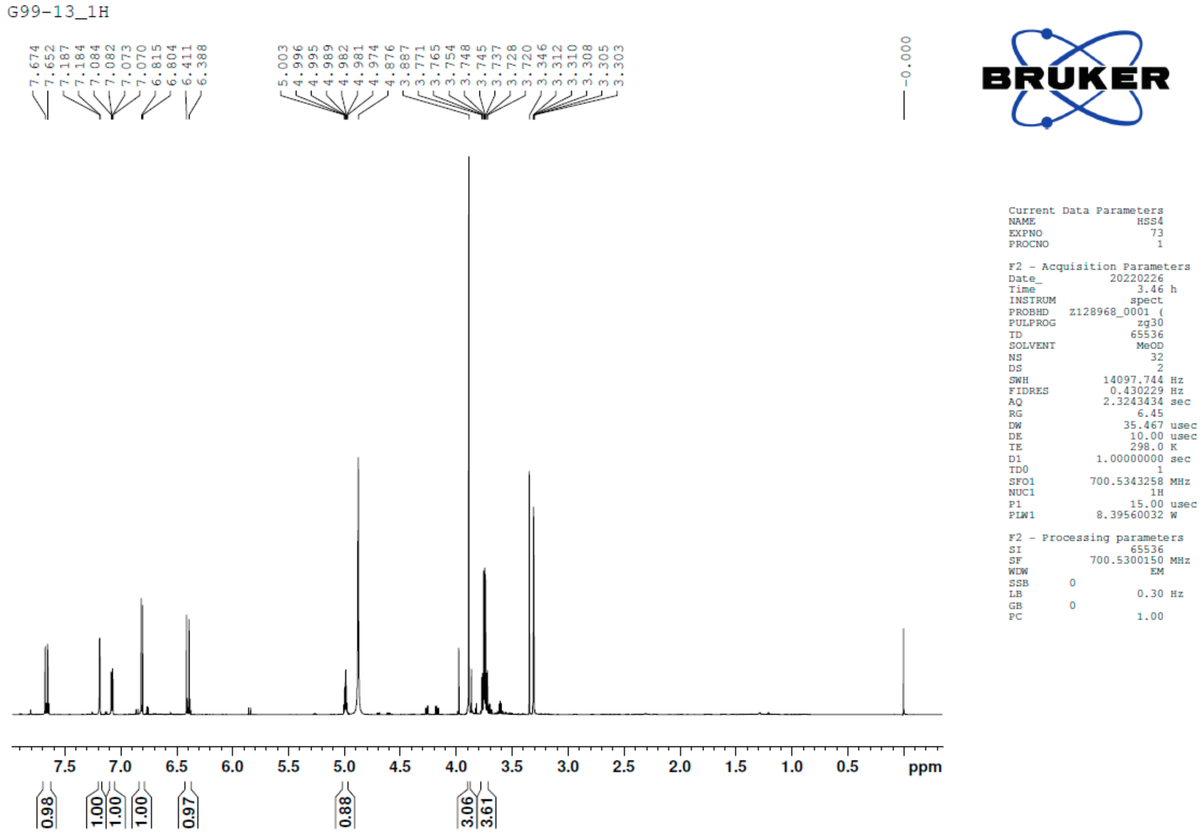

Figure S16. ^{1}H-NMR spectrum (CD_3OD, 700 MHz) of compound **8**

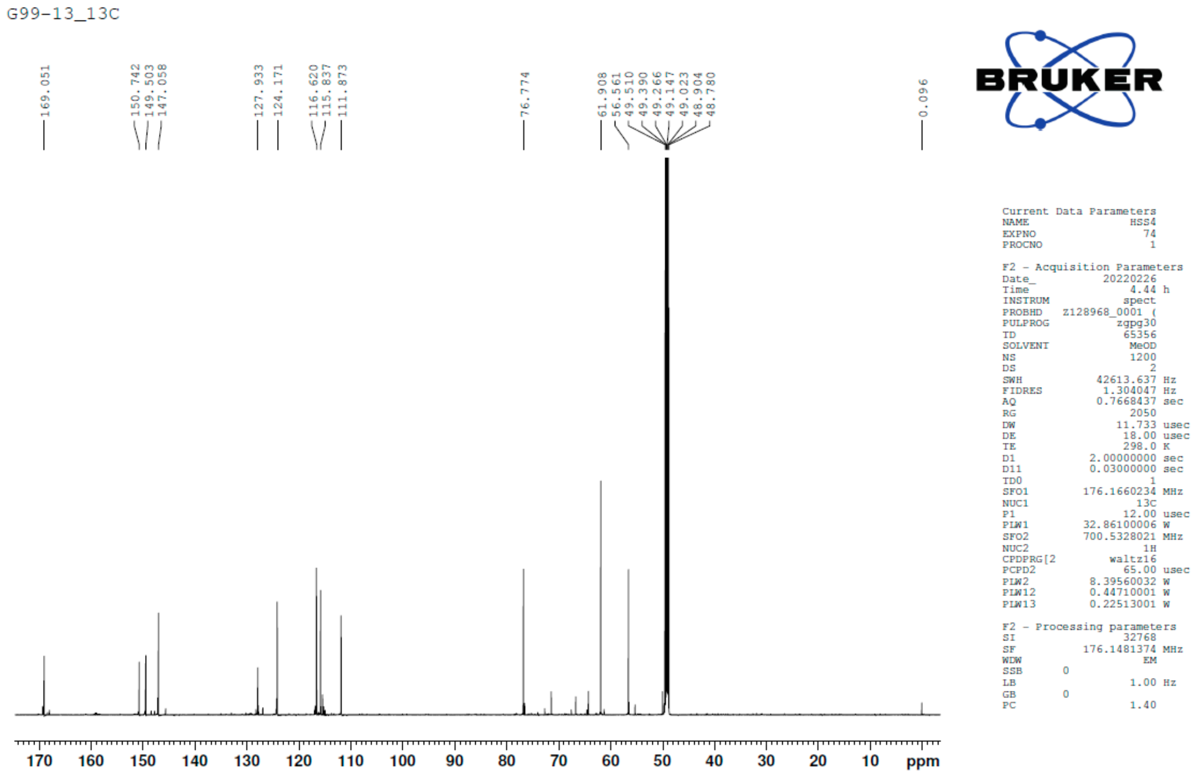

Figure S17. ^{13}C-NMR spectrum (CD_3OD, 175 MHz) of compound **8**

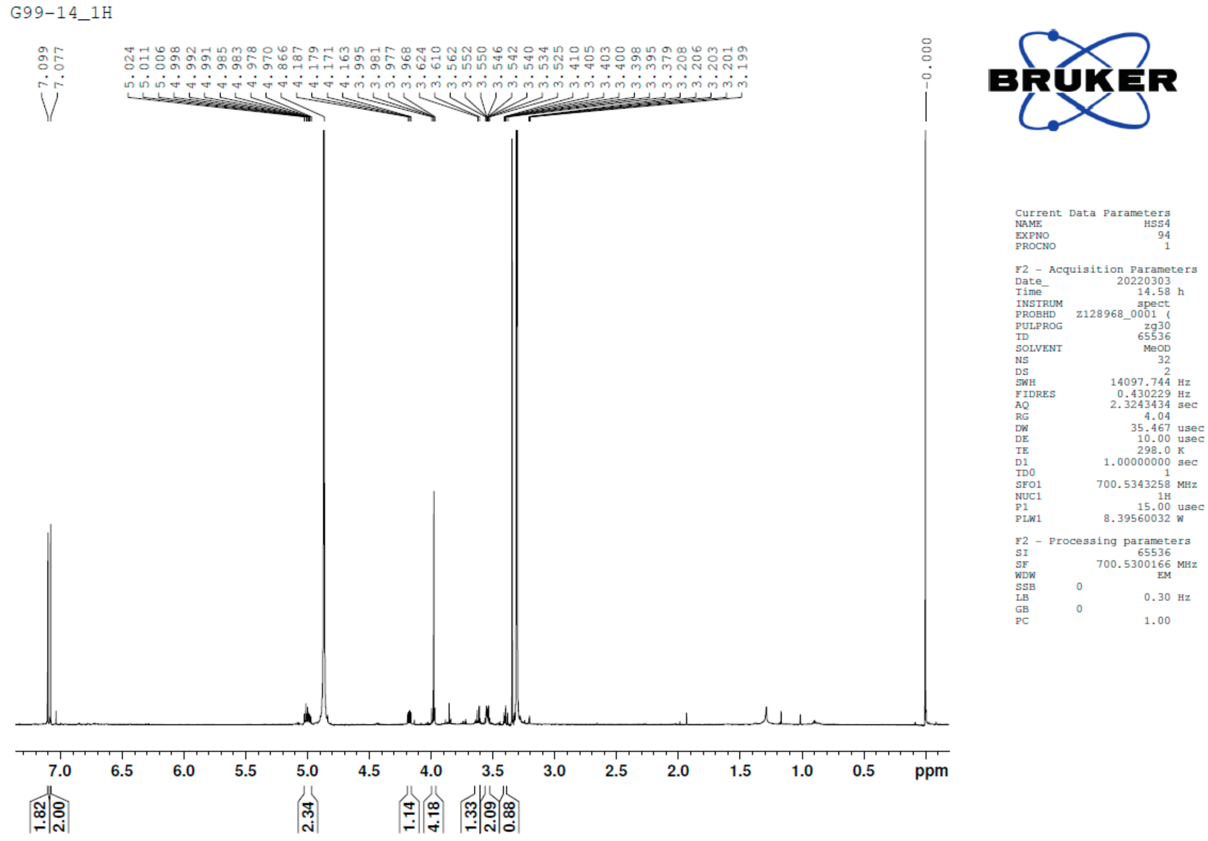

Figure S18. ^{1}H-NMR spectrum (CD_3OD, 700 MHz) of compound **9**

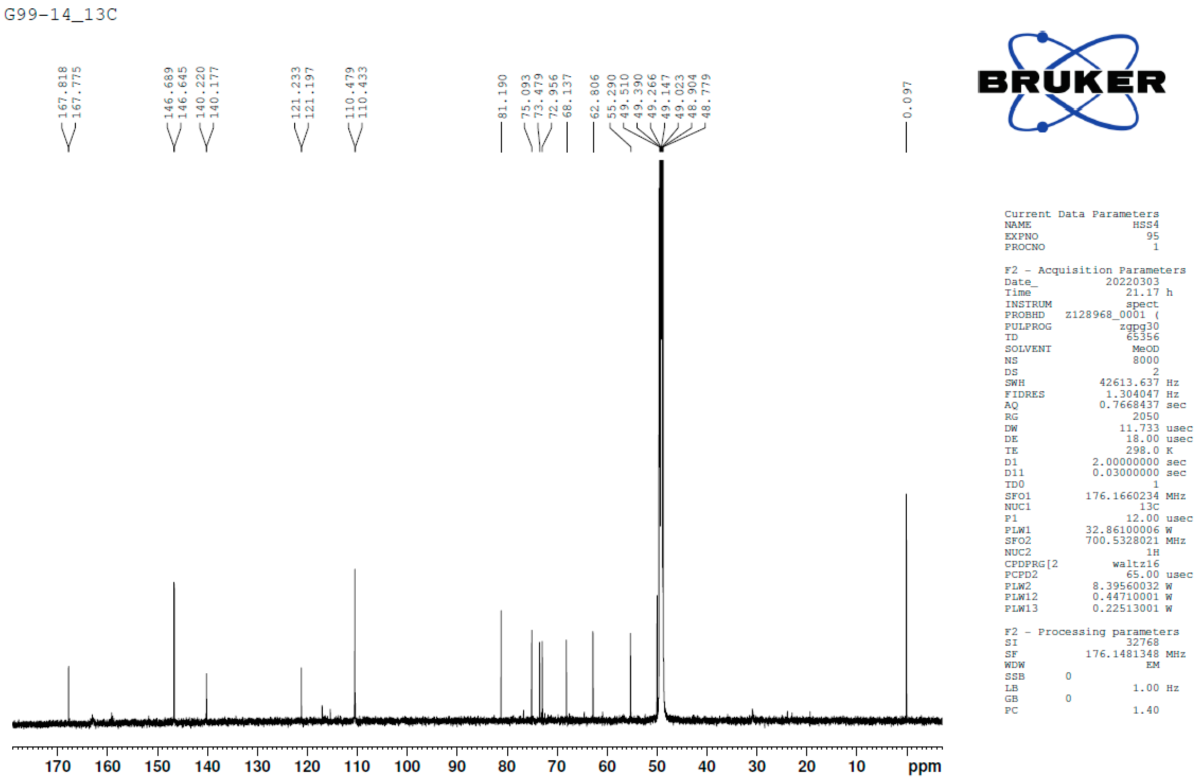

Figure S19. ^{13}C-NMR spectrum (CD_3OD, 175 MHz) of compound **9**

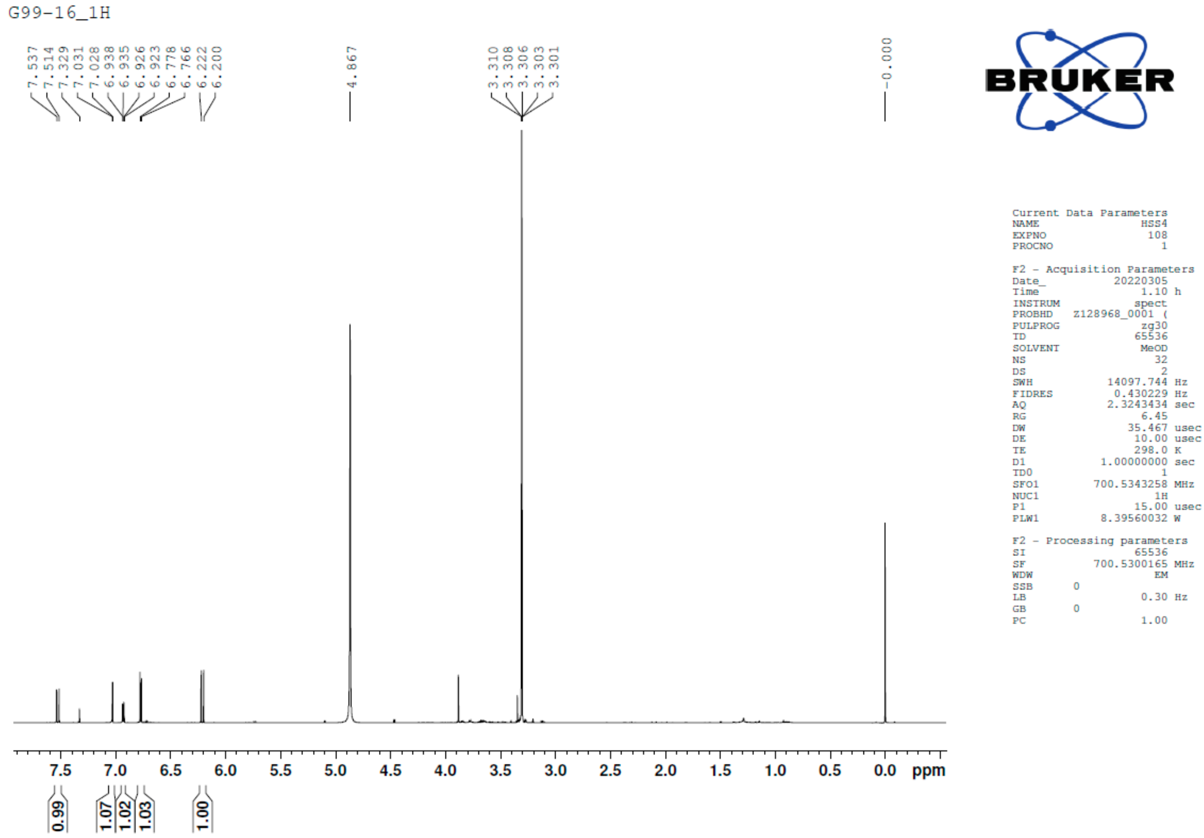

Figure S20. ^{1}H-NMR spectrum (CD_3OD, 700 MHz) of compound **10**

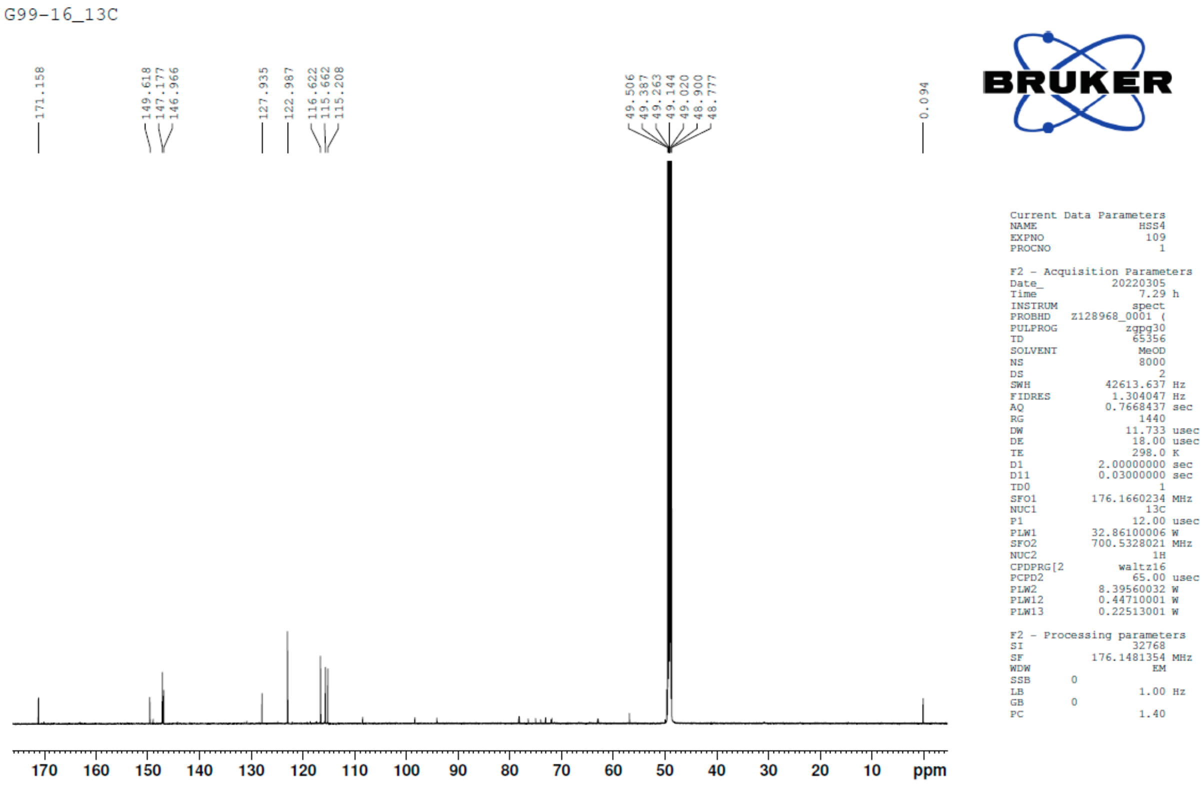

Figure S21. ^{13}C-NMR spectrum (CD_3OD, 175 MHz) of compound **10**

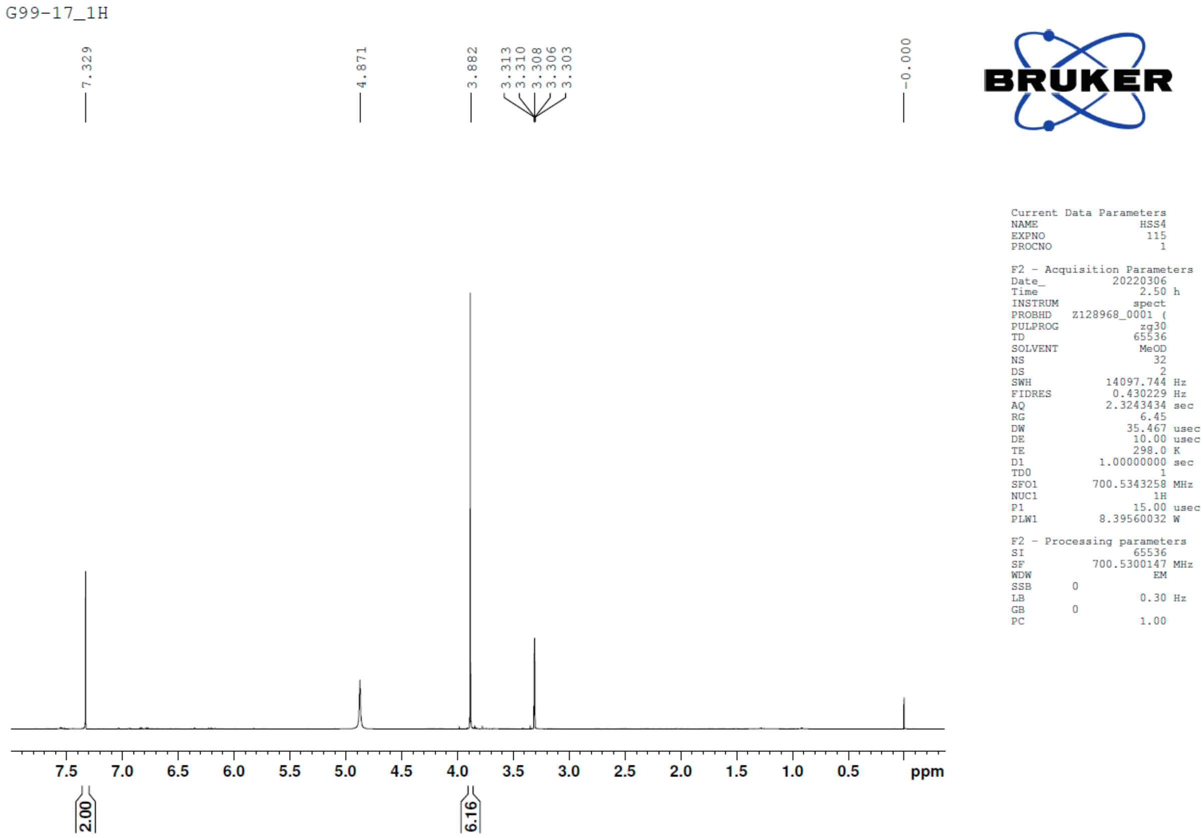

Figure S22. ^{1}H-NMR spectrum (CD_3OD, 700 MHz) of compound **11**

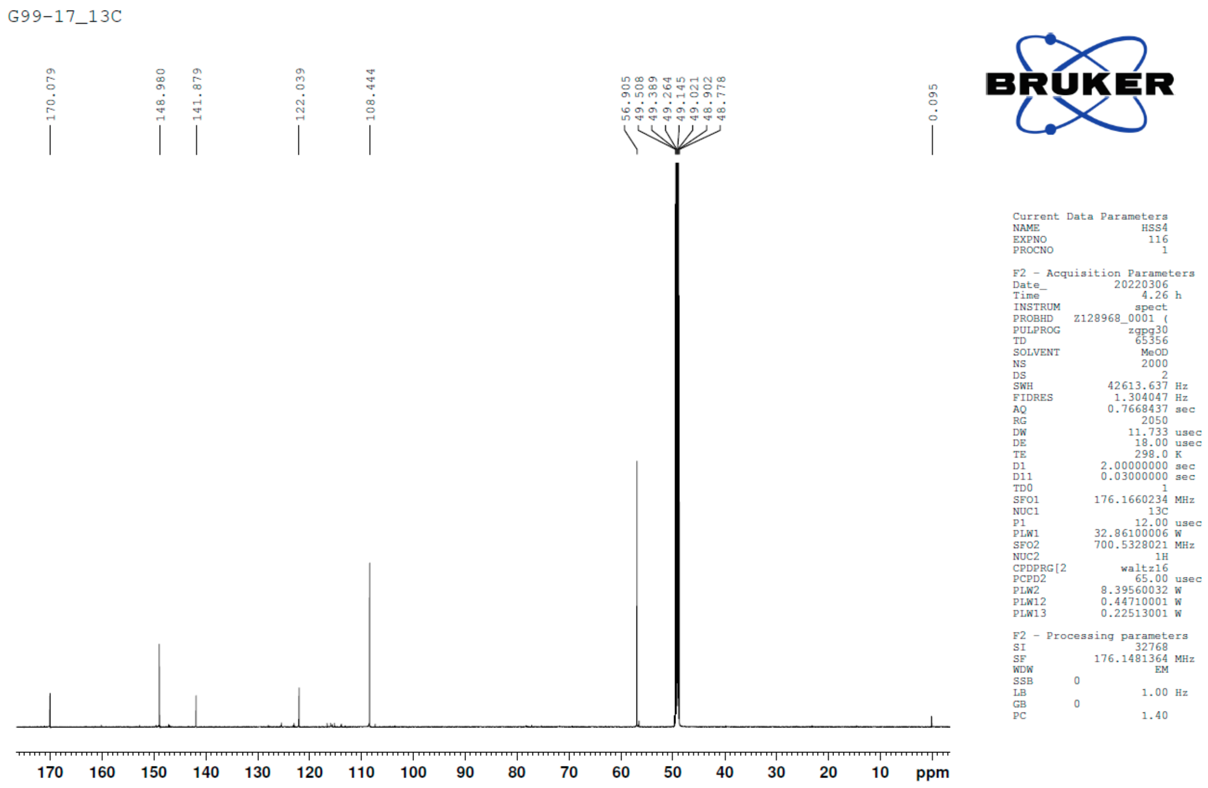

Figure S23. ^{13}C-NMR spectrum (CD_3OD, 175 MHz) of compound **11**

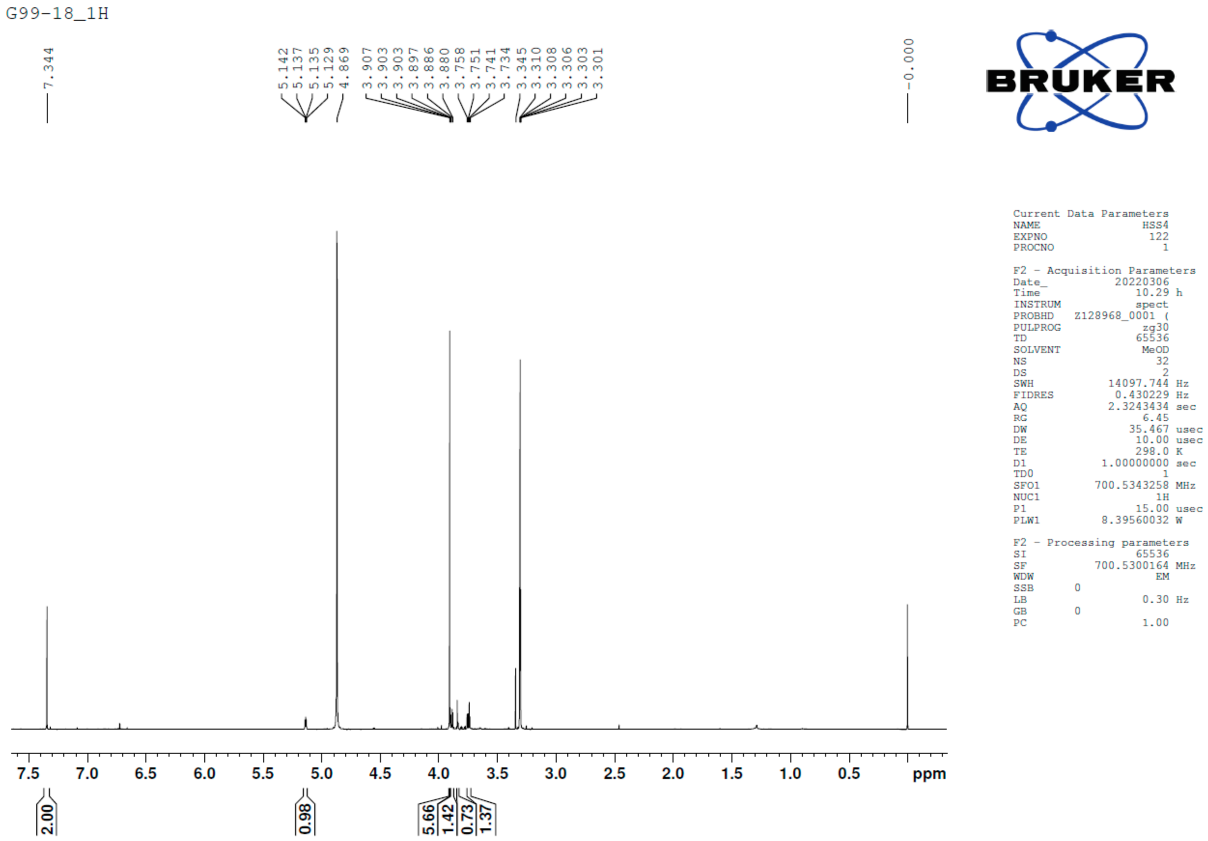

Figure S24. ^{1}H-NMR spectrum (CD_3OD, 700 MHz) of compound **12**

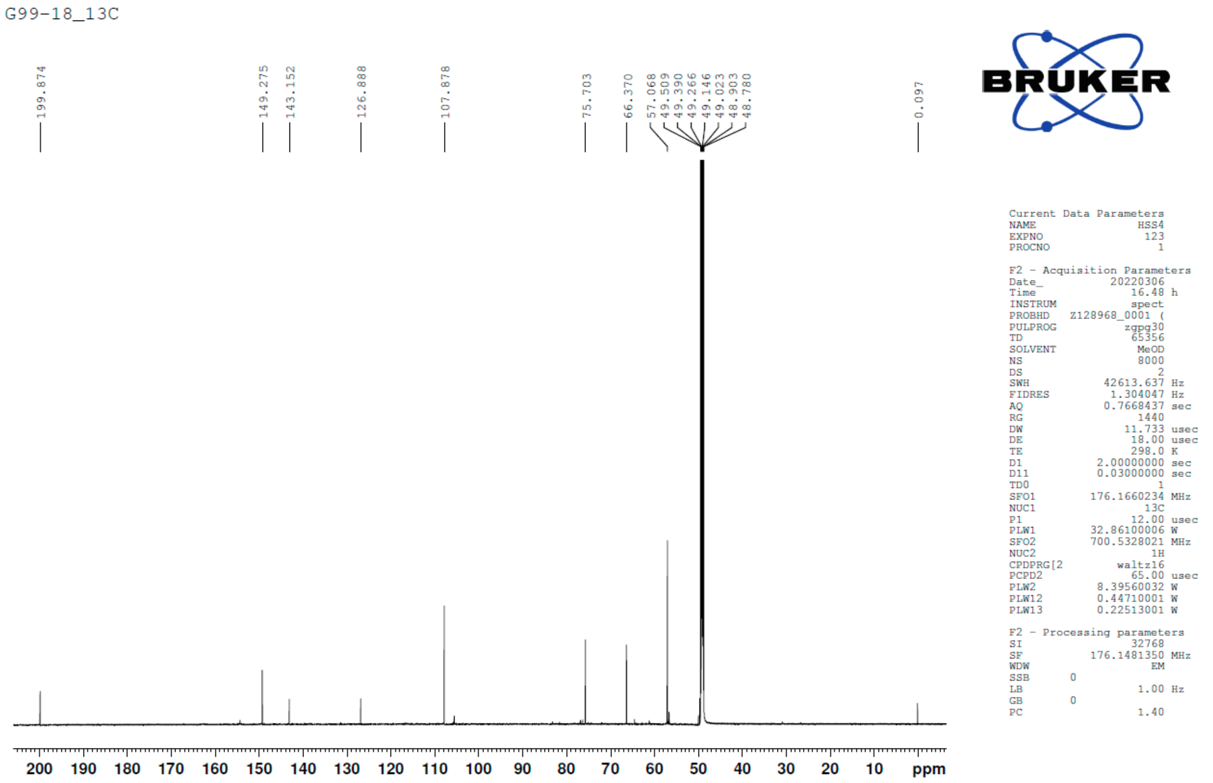

Figure S25. ^{13}C-NMR spectrum (CD_3OD, 175 MHz) of compound **12**

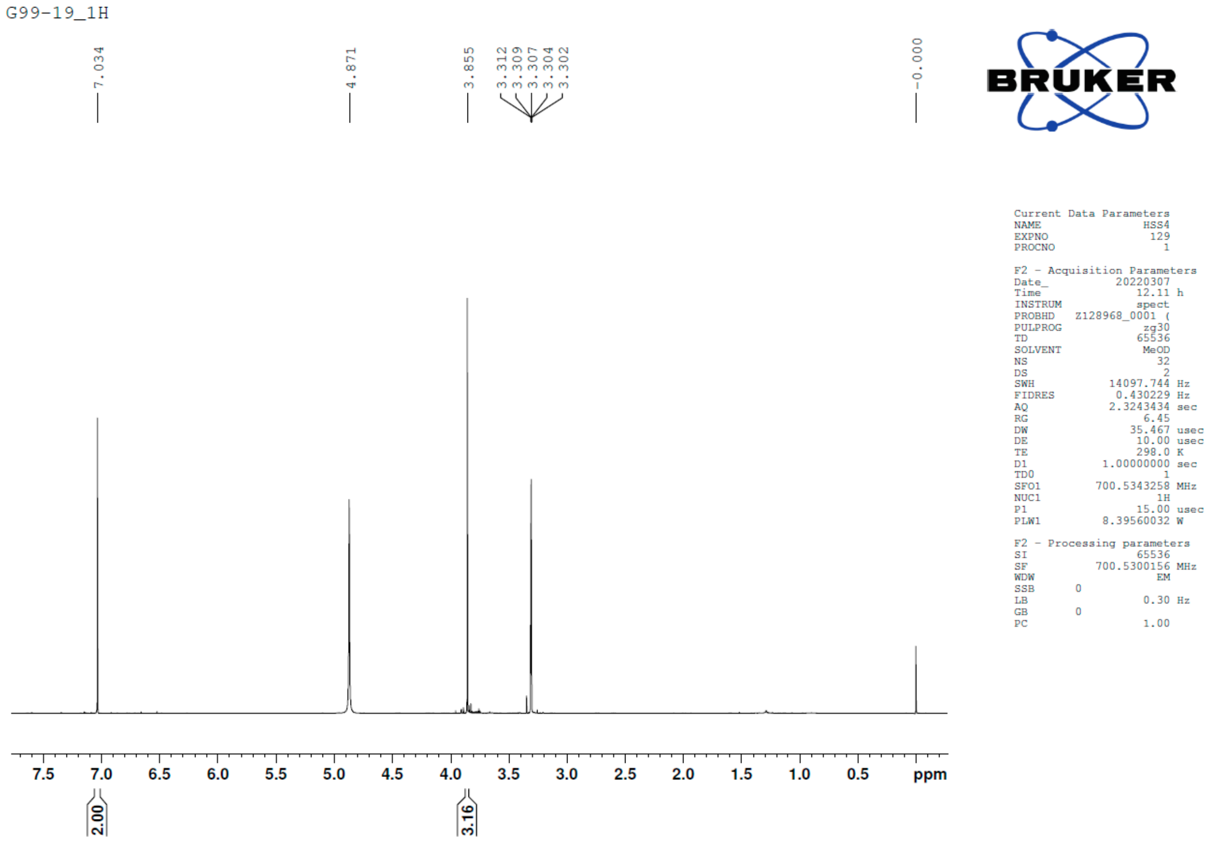

Figure S26. ^{1}H-NMR spectrum (CD_3OD, 700 MHz) of compound **13**

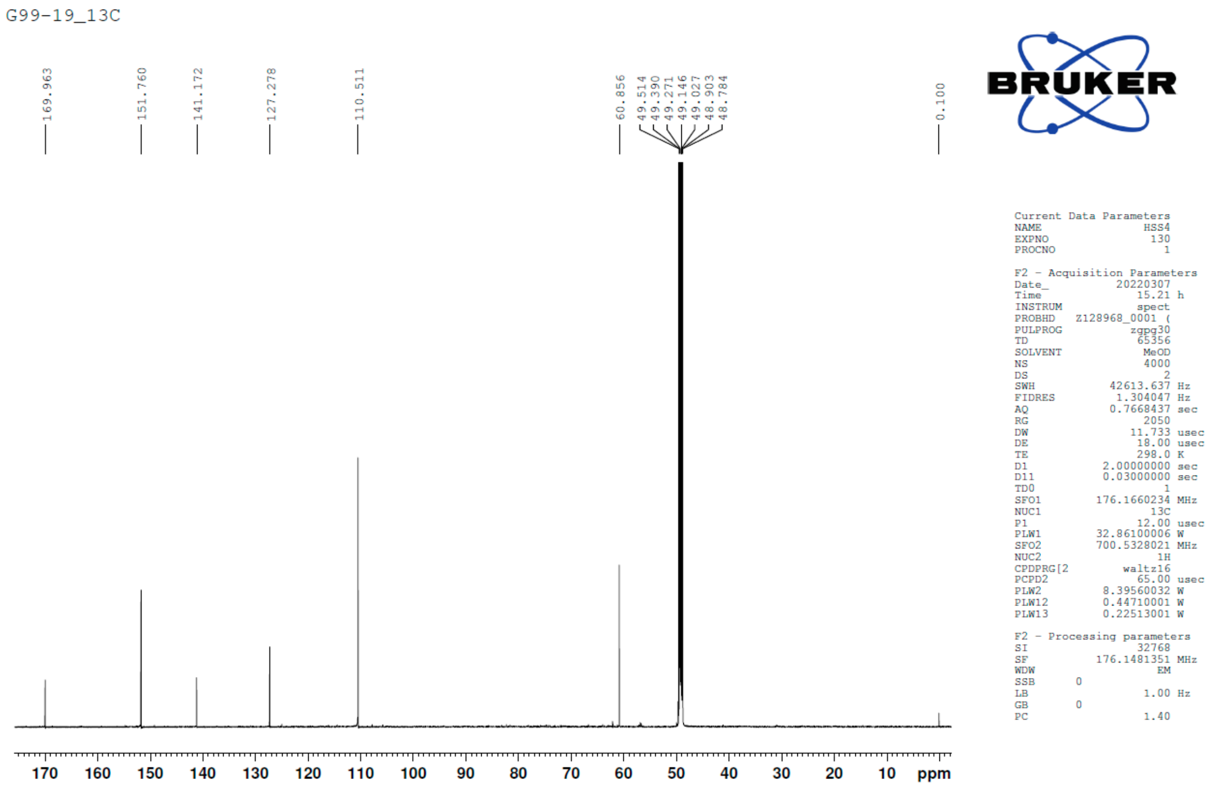

Figure S27. ^{13}C-NMR spectrum (CD_3OD, 175 MHz) of compound **13**

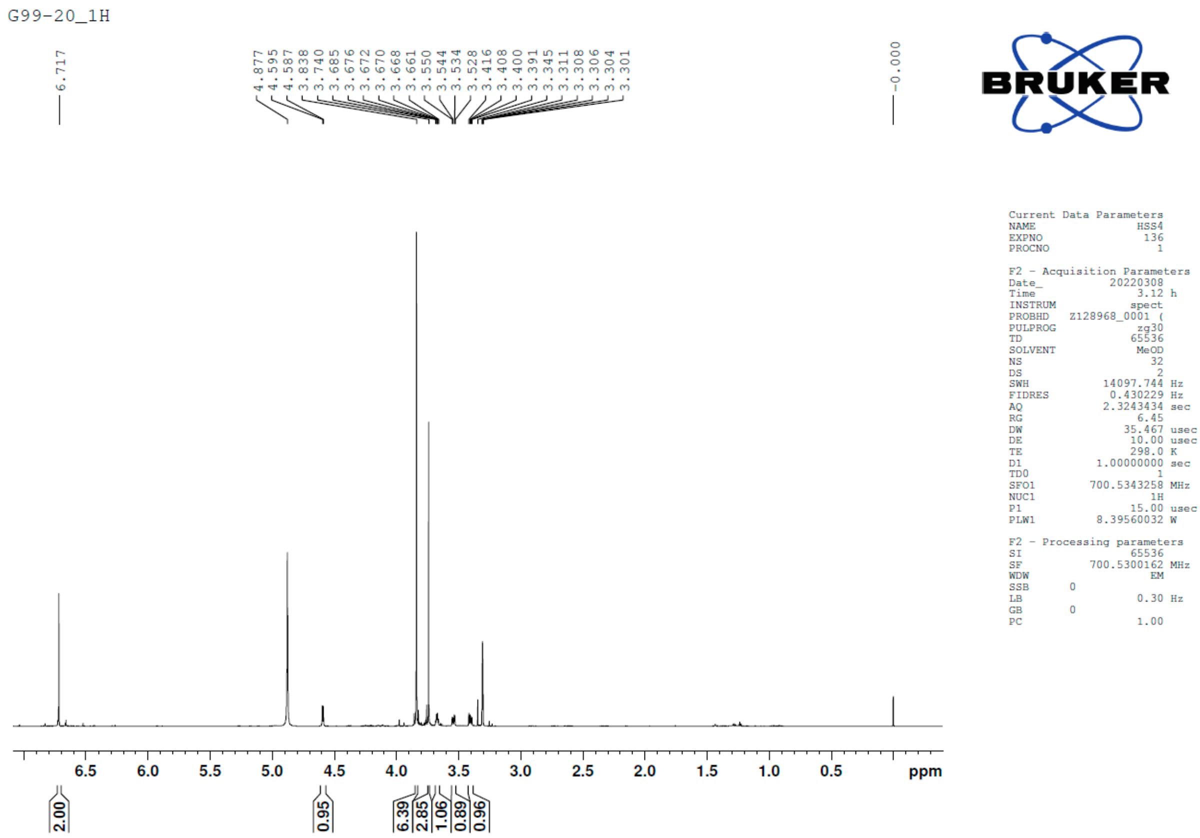

Figure S28. ^{1}H-NMR spectrum (CD_3OD, 700 MHz) of compound **14**

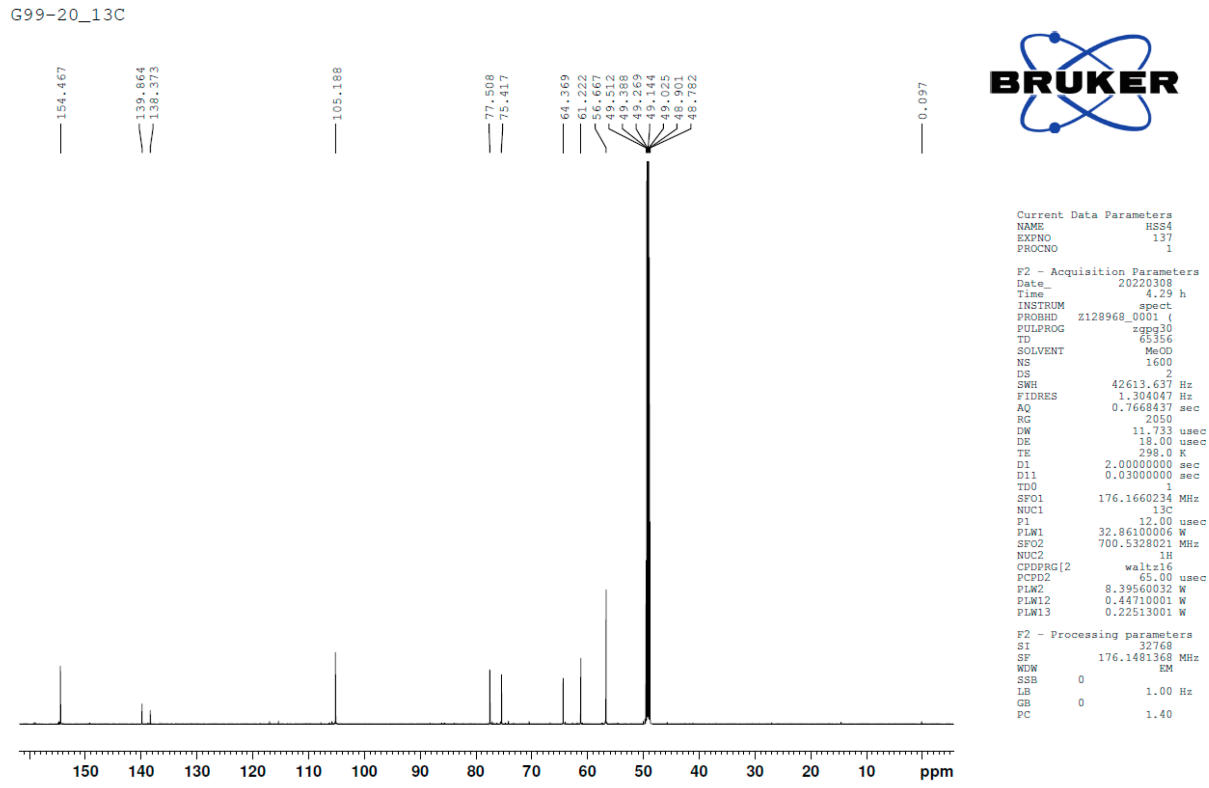

Figure S29. ^{13}C-NMR spectrum (CD_3OD, 175 MHz) of compound **14**

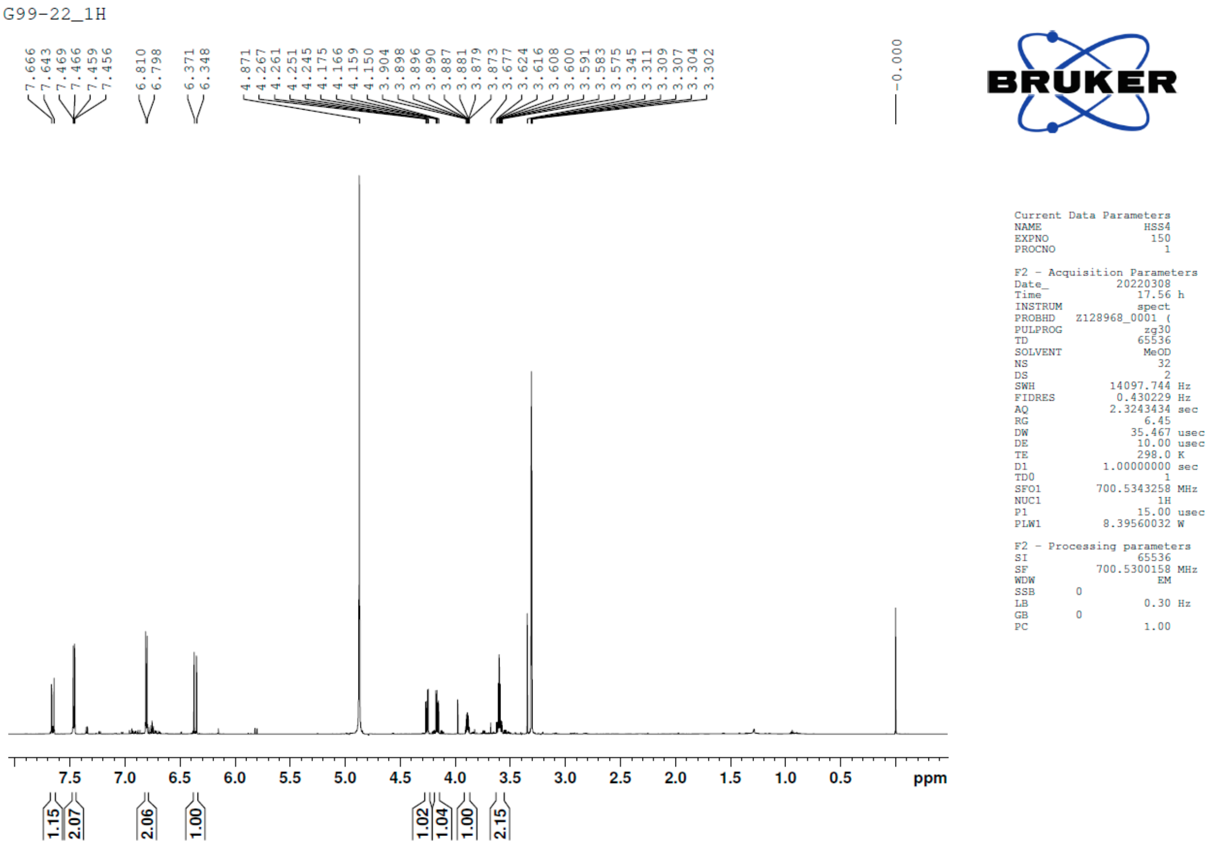

Figure S30. ^{1}H-NMR spectrum (CD_3OD, 700 MHz) of compound **15**

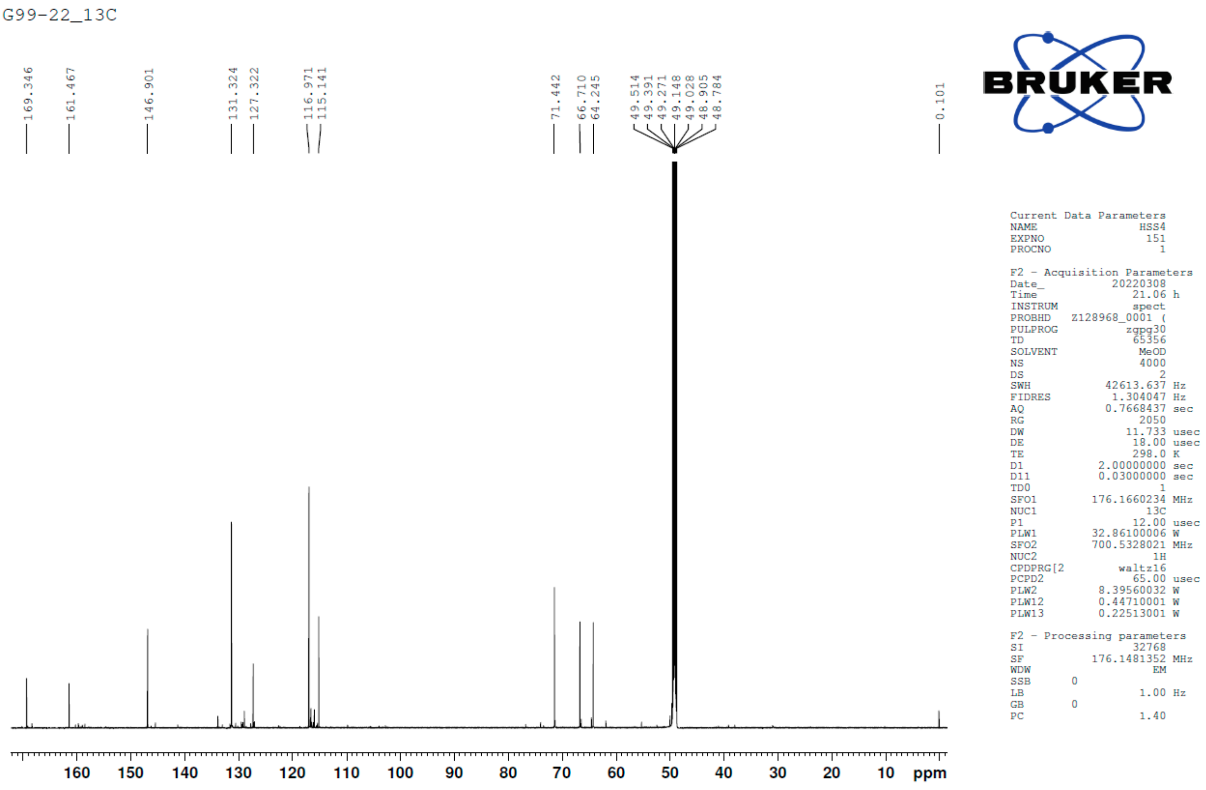

Figure S31. ^{13}C-NMR spectrum (CD_3OD, 175 MHz) of compound **15**

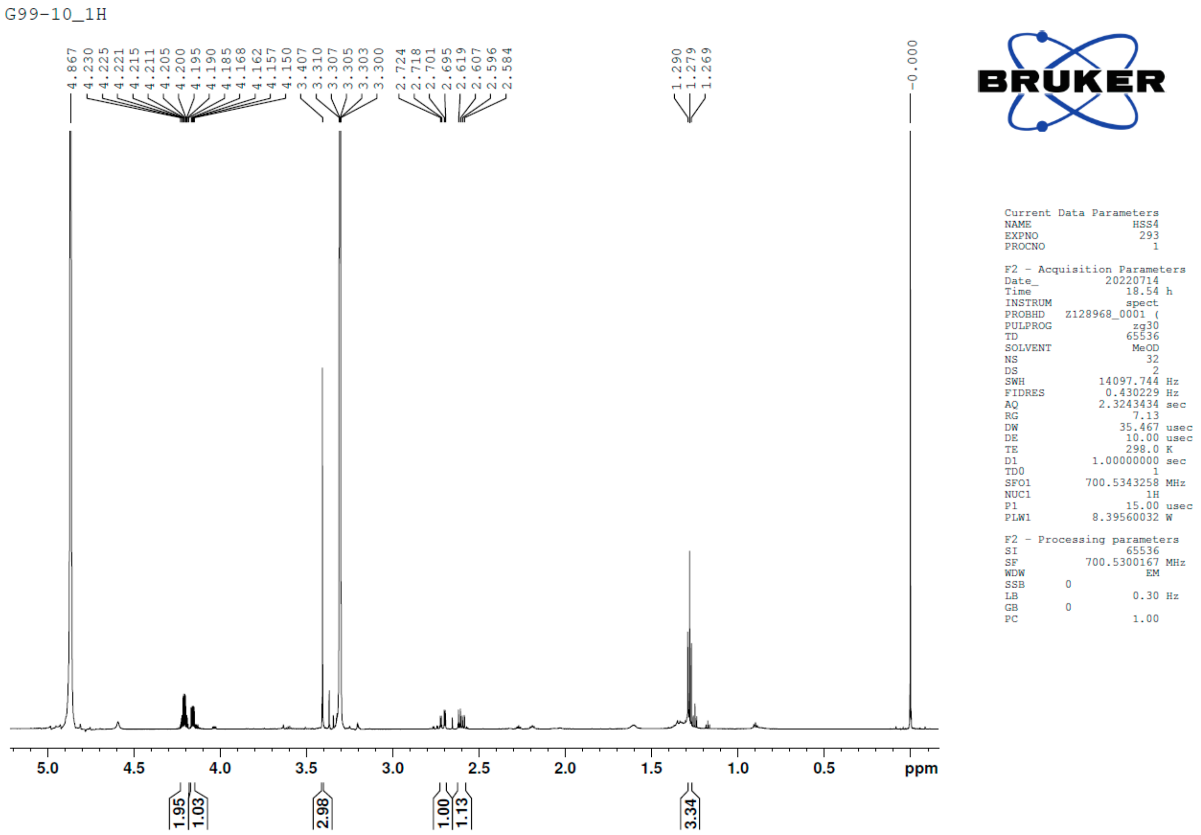

Figure S32. ^{1}H-NMR spectrum (CD_3OD, 700 MHz) of compound **16**

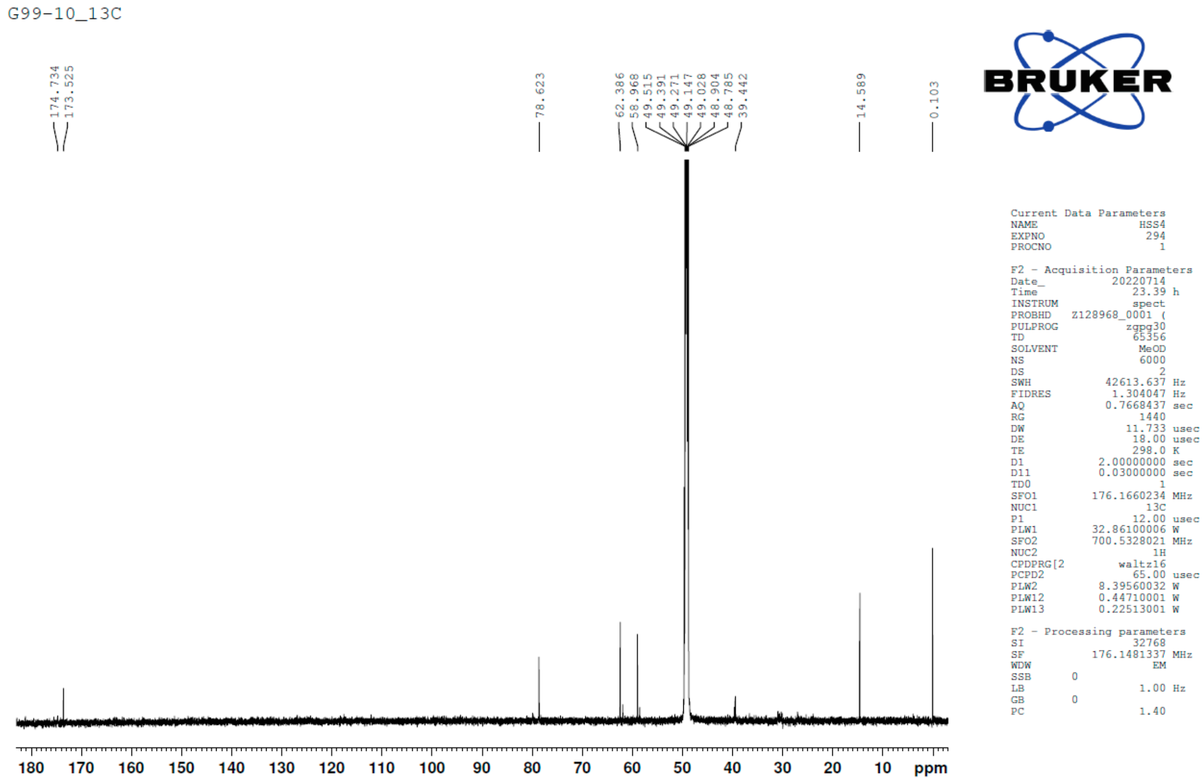

Figure S33. ^{13}C-NMR spectrum (CD_3OD, 175 MHz) of compound **16**

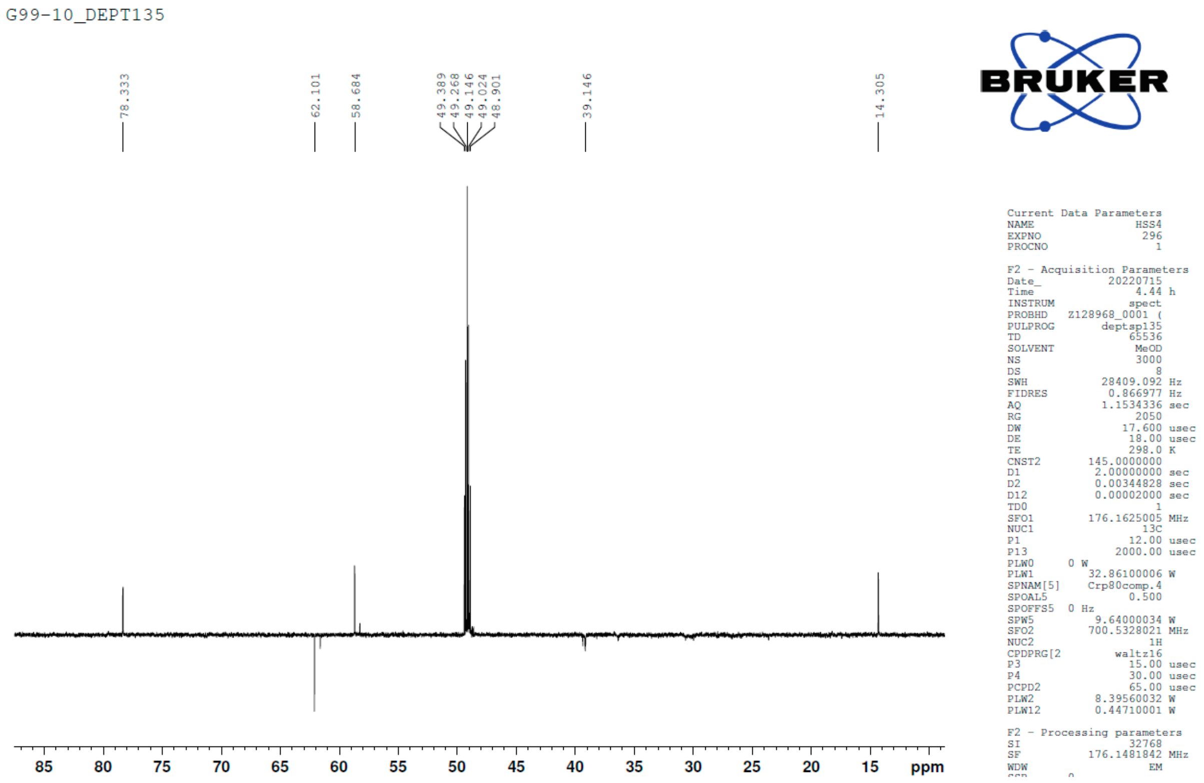

Figure S34. DEPT135 spectrum of compound **16**

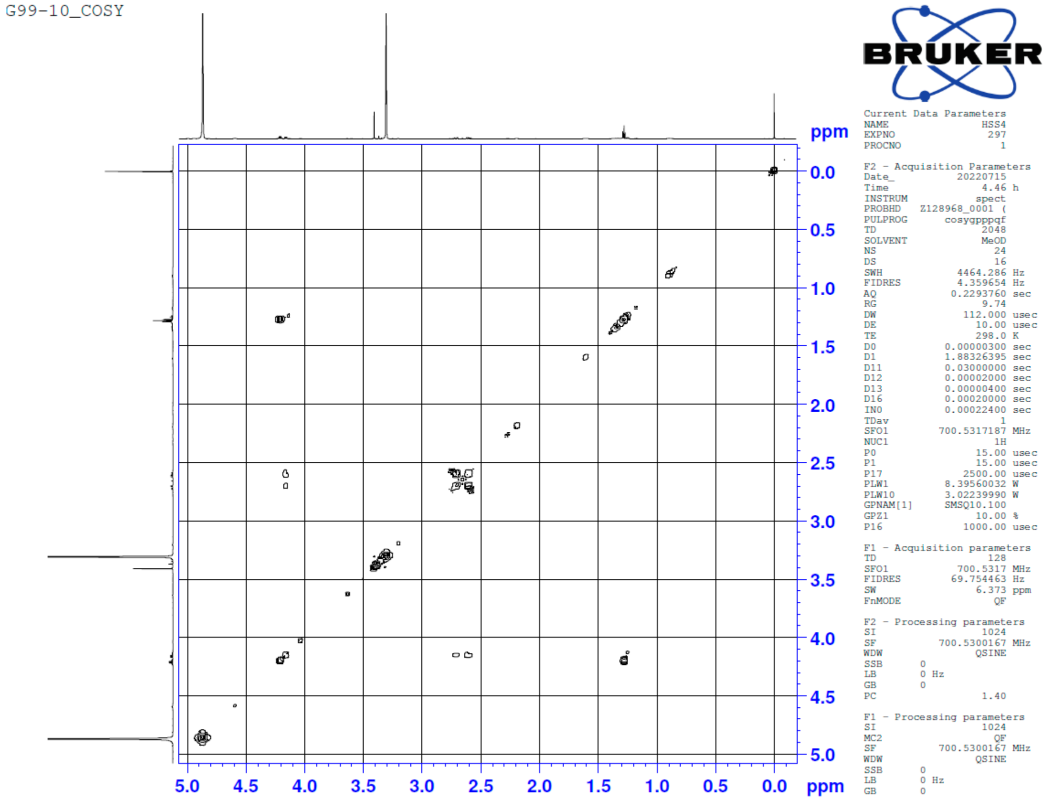

Figure S35. ^{1}H-^{1}H COSY spectrum of compound **16**

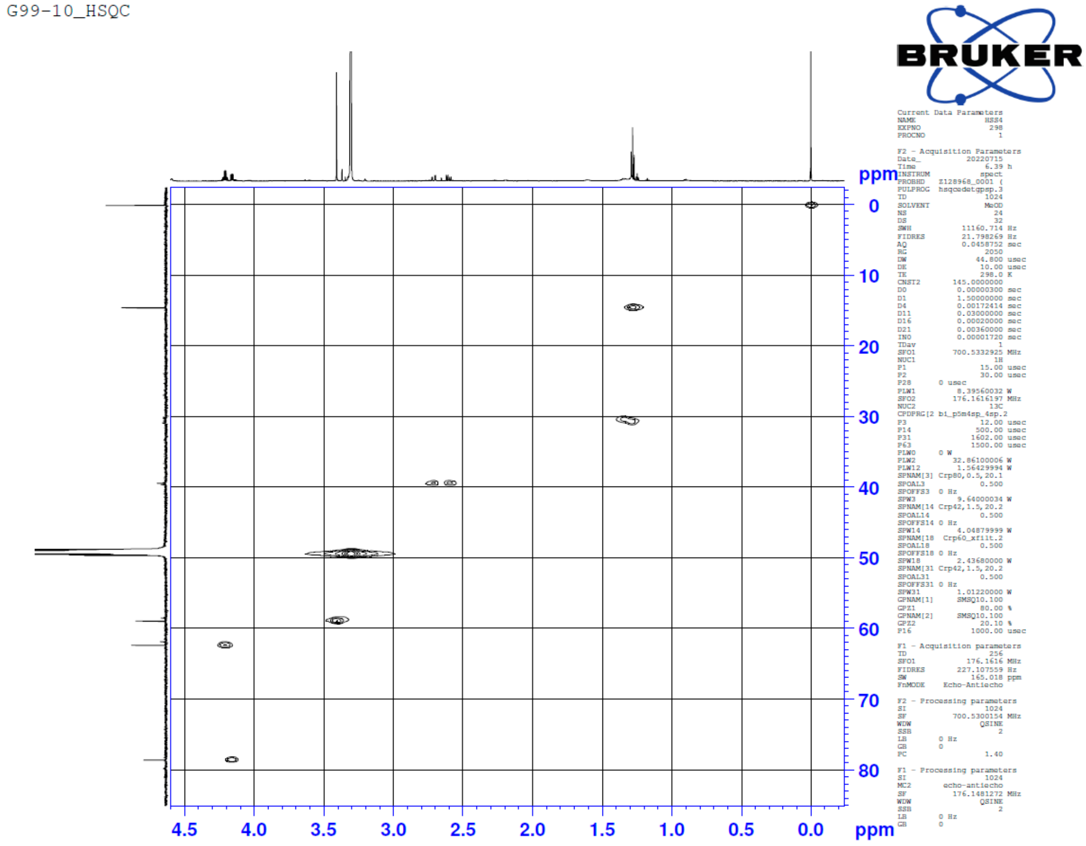

Figure S36. HSQC spectrum of compound **16**

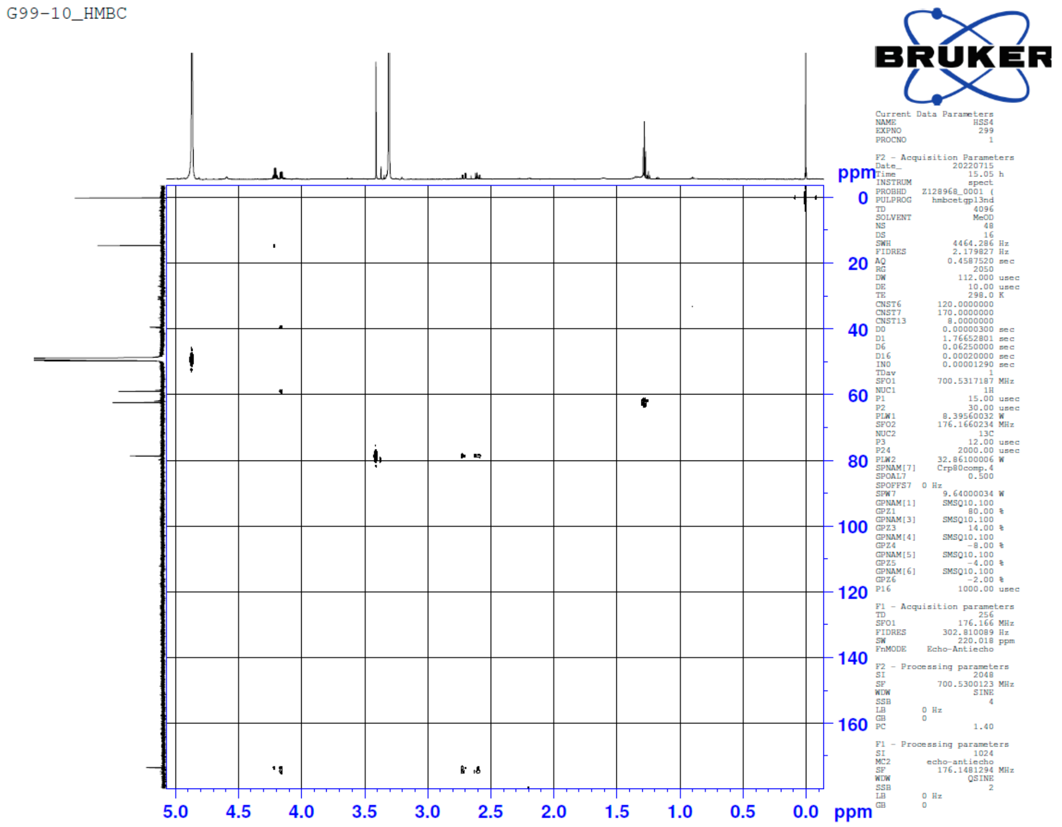

Figure S37. HMBC spectrum of compound **16**

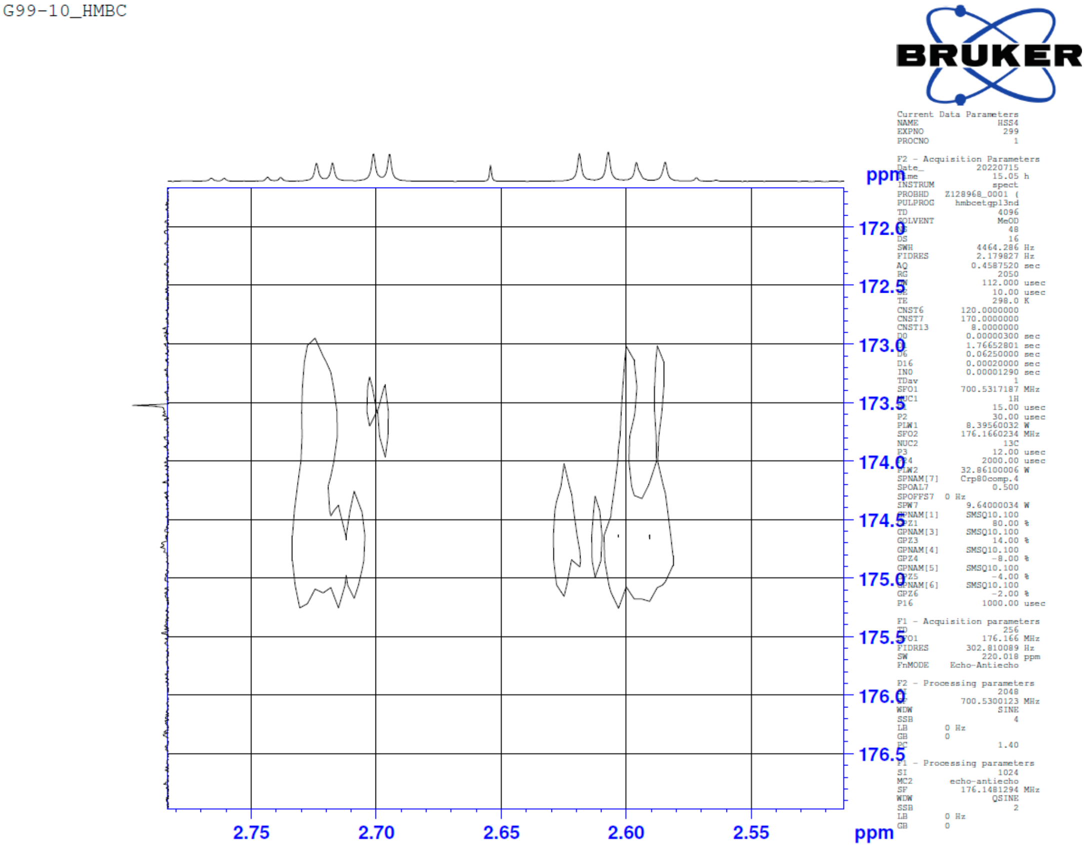

Figure S38. Expansion of HMBC spectrum of compound **16**

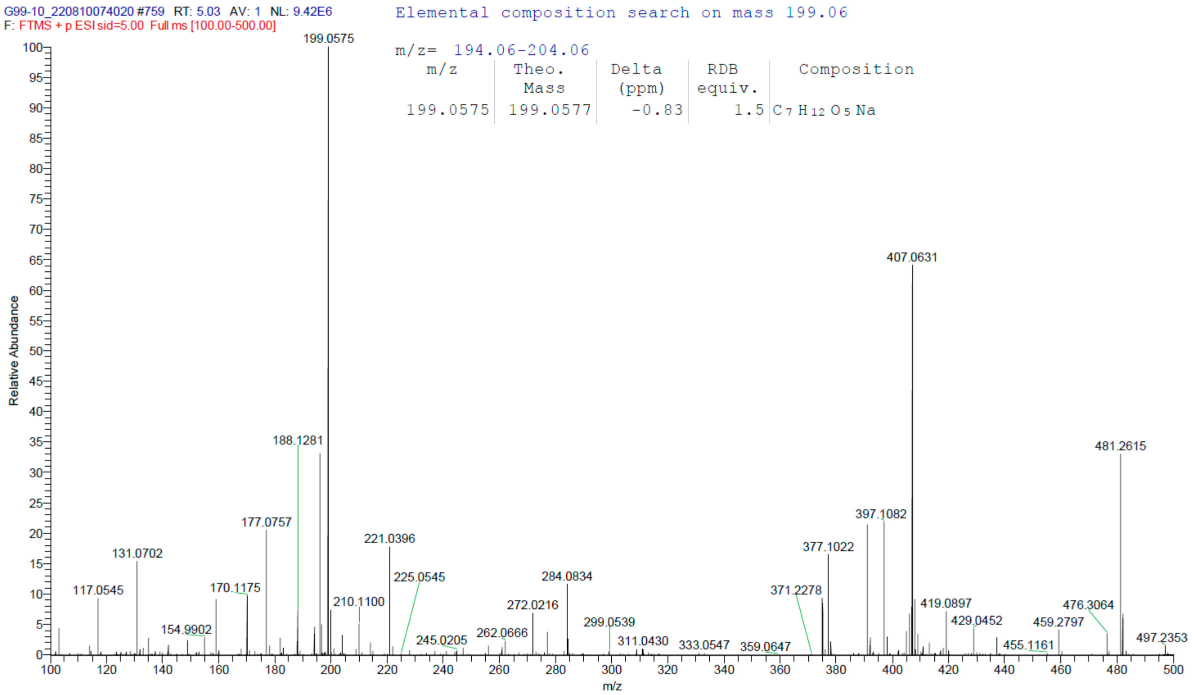

Figure S39. HRESIMS spectrum of compound **16**

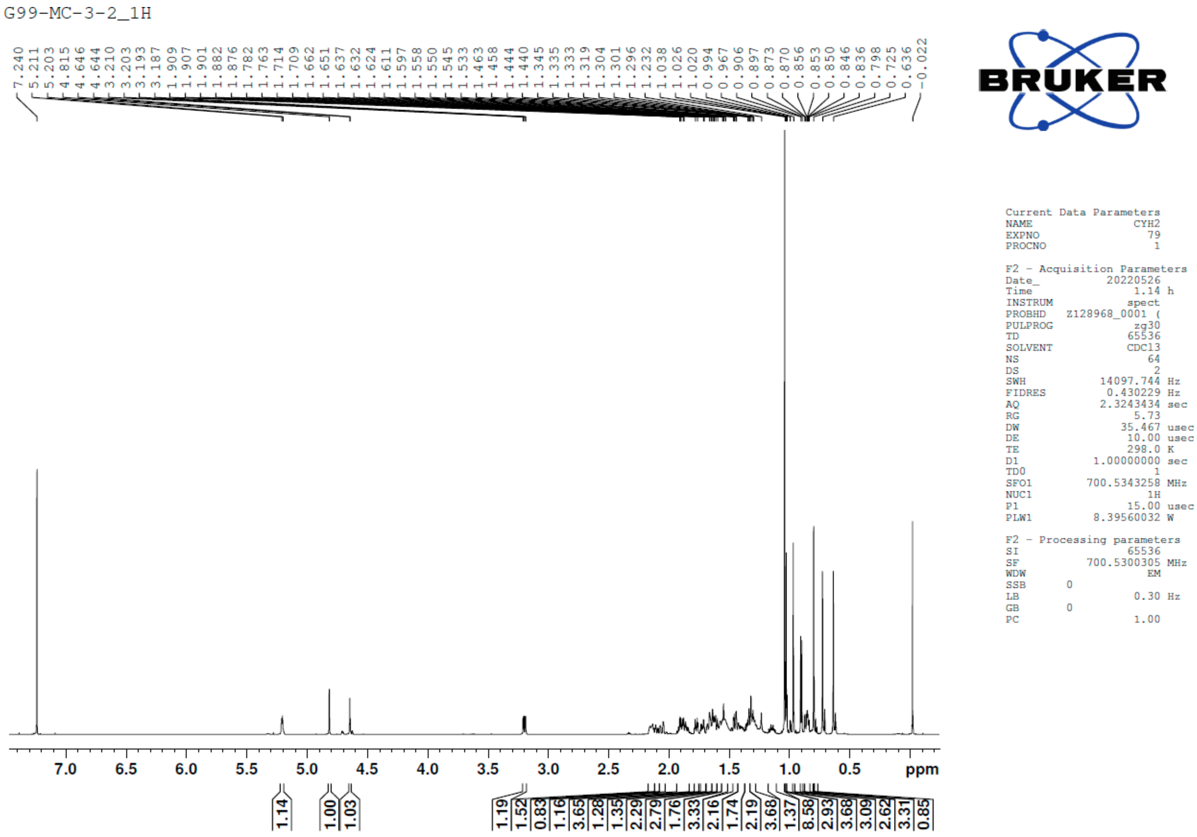

Figure S40. ^{1}H-NMR spectrum ($CDCl_3$, 700 MHz) of compound **17**

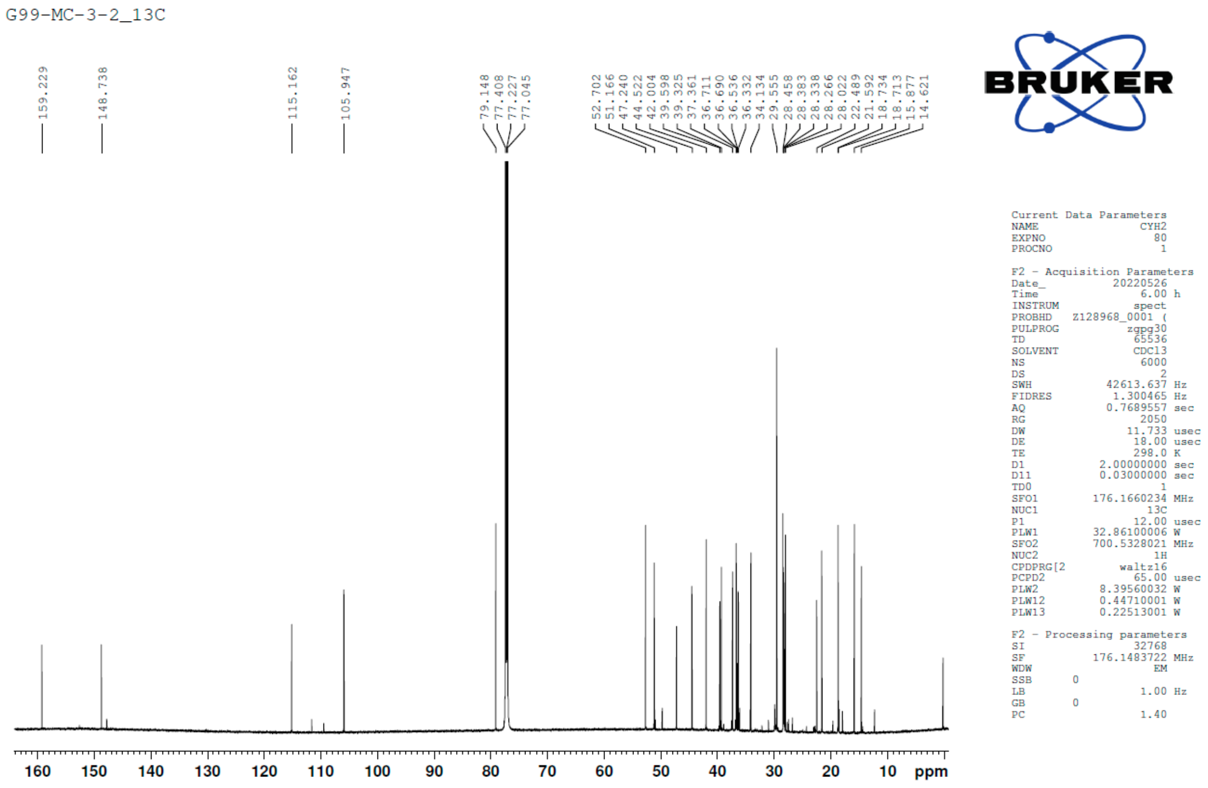

Figure S41. ^{13}C-NMR spectrum ($CDCl_3$, 175 MHz) of compound **17**

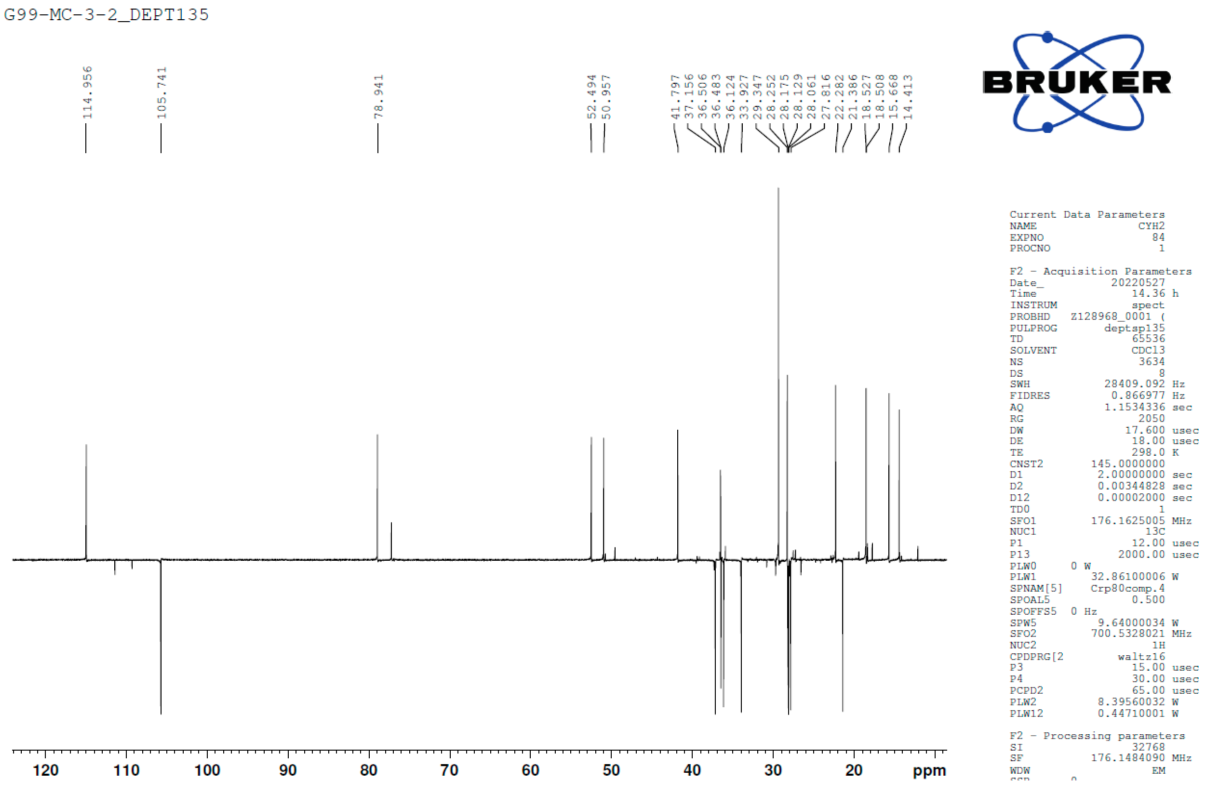

Figure S42. DEPT135 spectrum of compound **17**

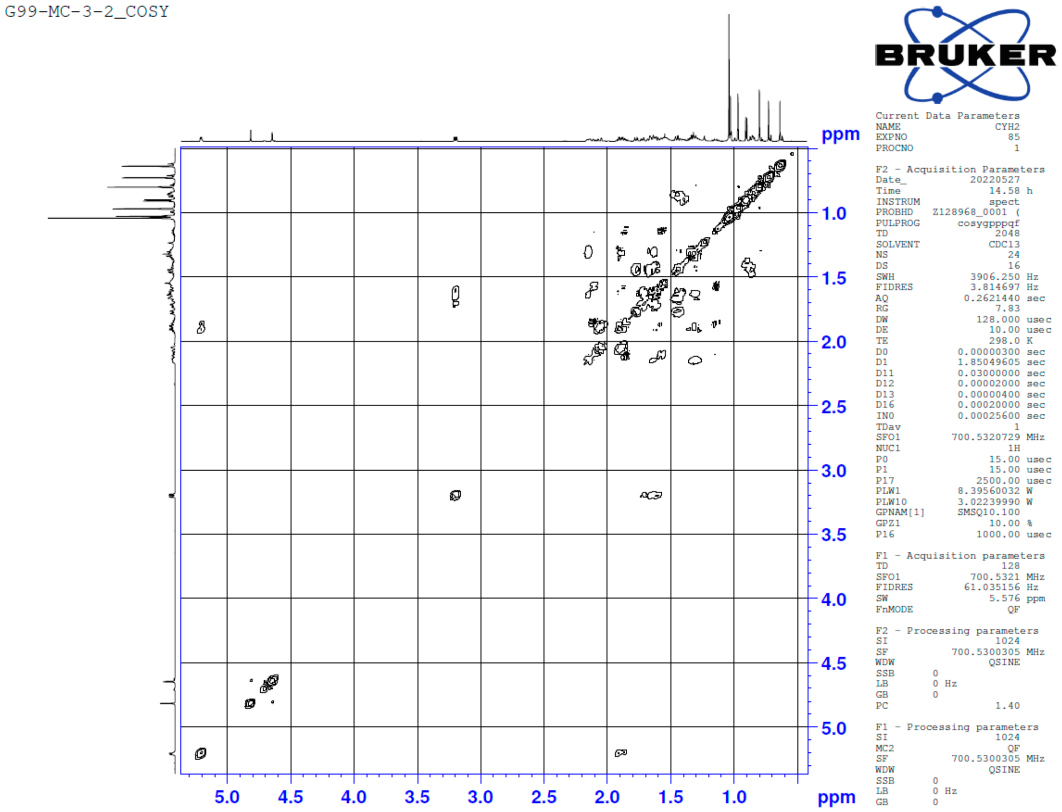

Figure S43. ^{1}H-^{1}H COSY spectrum of compound **17**

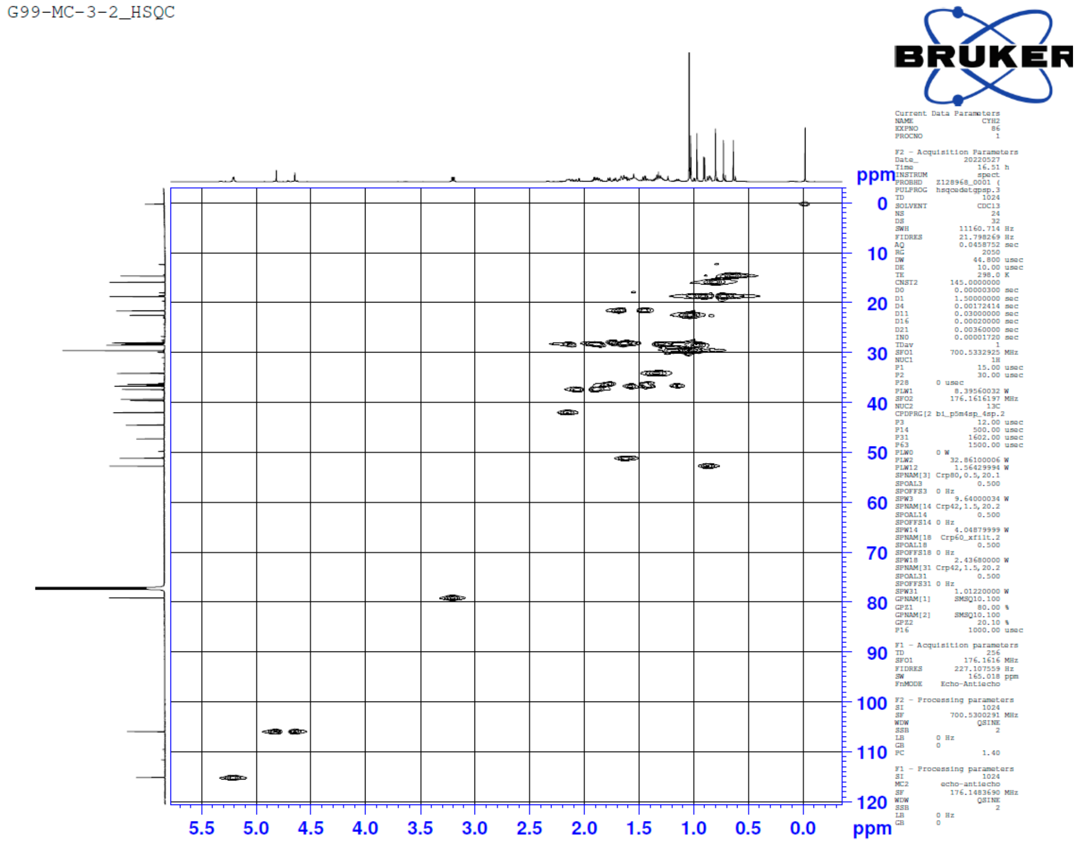

Figure S44. HSQC spectrum of compound **17**

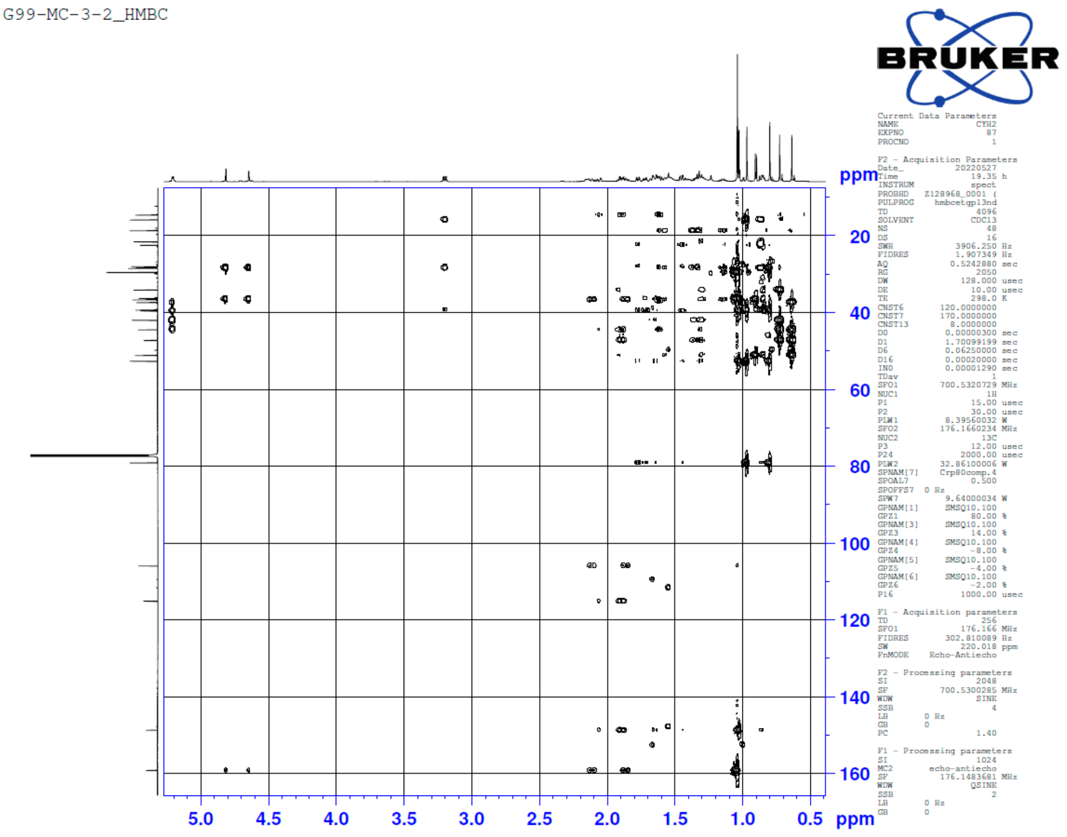

Figure S45. HMBC spectrum of compound **17**

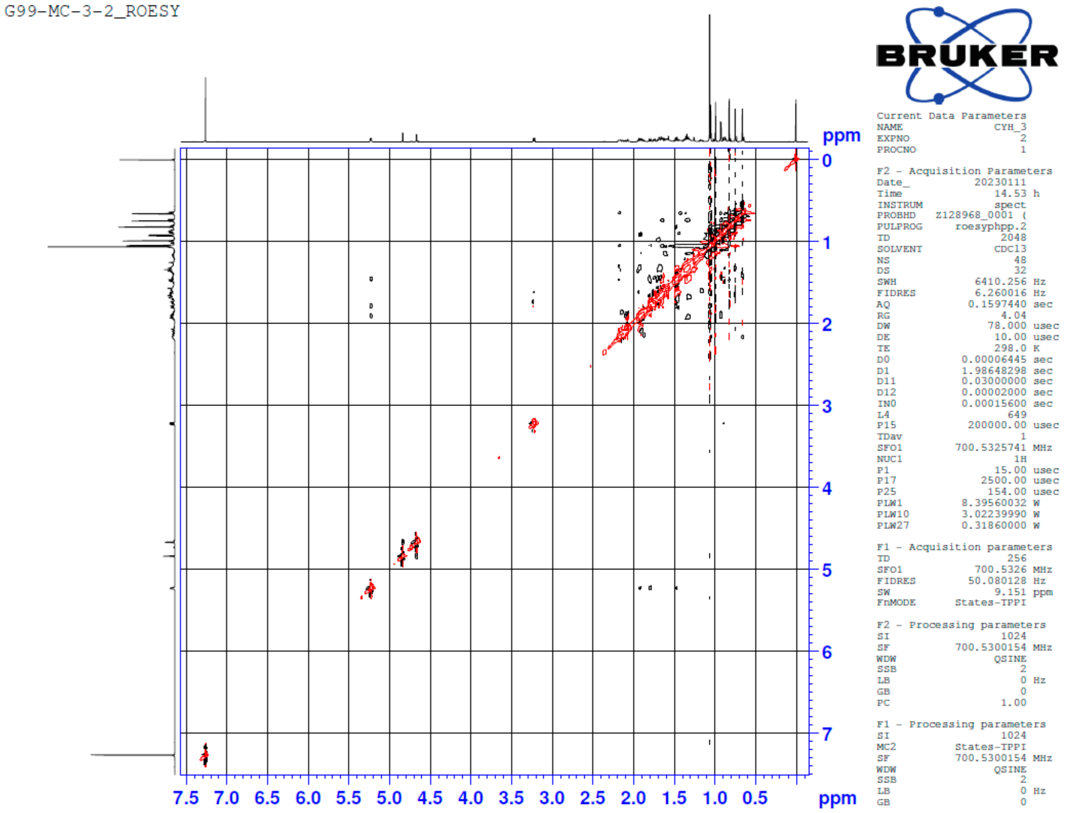

Figure S46. ROESY spectrum of compound **17**

Light Source	Na
Monitor wavelength	589 nm
D.I.T.	5 sec
No. of cycle	5
Cycle interval	0 sec
Temp. Monitor	Cell
Temp. Corr. Factor	None
Aperture(S)	8.0mm
Aperture(L)	Auto
Mode	Specific O.R.
Path Length	50 mm
Concentration	0.114286 w/v%
Water content of sample	0 %
Factor	1

		No.	Sample No.	Mode	Calc. Data	Meas. Data	Monitor(deg)	PMT Voltage[V]	Temperature(C)
1	*	1	G99-MC-3P-1	Specific O.R.	44.2049	0.0253	0.0233	305	25.17
2	*	2	G99-MC-3P-2	Specific O.R.	48.2299	0.0276	0.0256	303	25.17
3	*	3	G99-MC-3P-3	Specific O.R.	50.6799	0.0290	0.0270	305	25.18
4	*	4	G99-MC-3P-4	Specific O.R.	39.8299	0.0228	0.0208	305	25.18
5	*	5	G99-MC-3P-5	Specific O.R.	49.1049	0.0281	0.0261	303	25.19
6	*	6	Avg.		46.4099				
7		7	S.D		4.3865				
8		8	C.V		9.4517				

Figure S47. Specific optical rotation of compound **17**

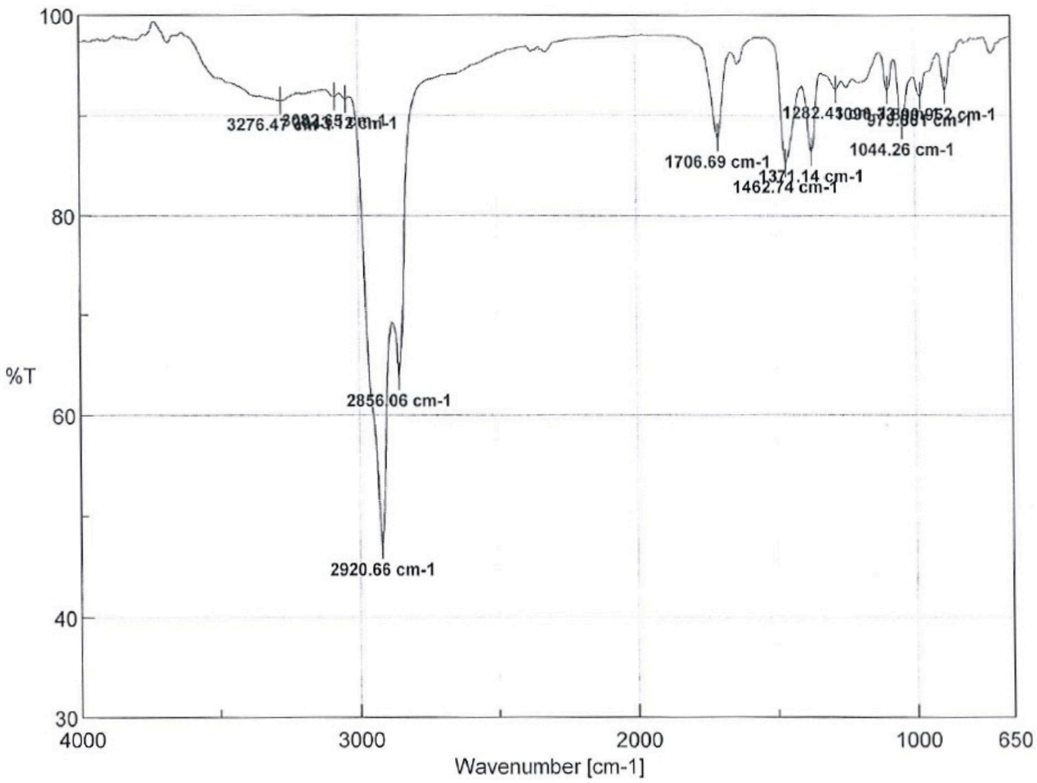

Figure S48. IR spectrum of compound **17**

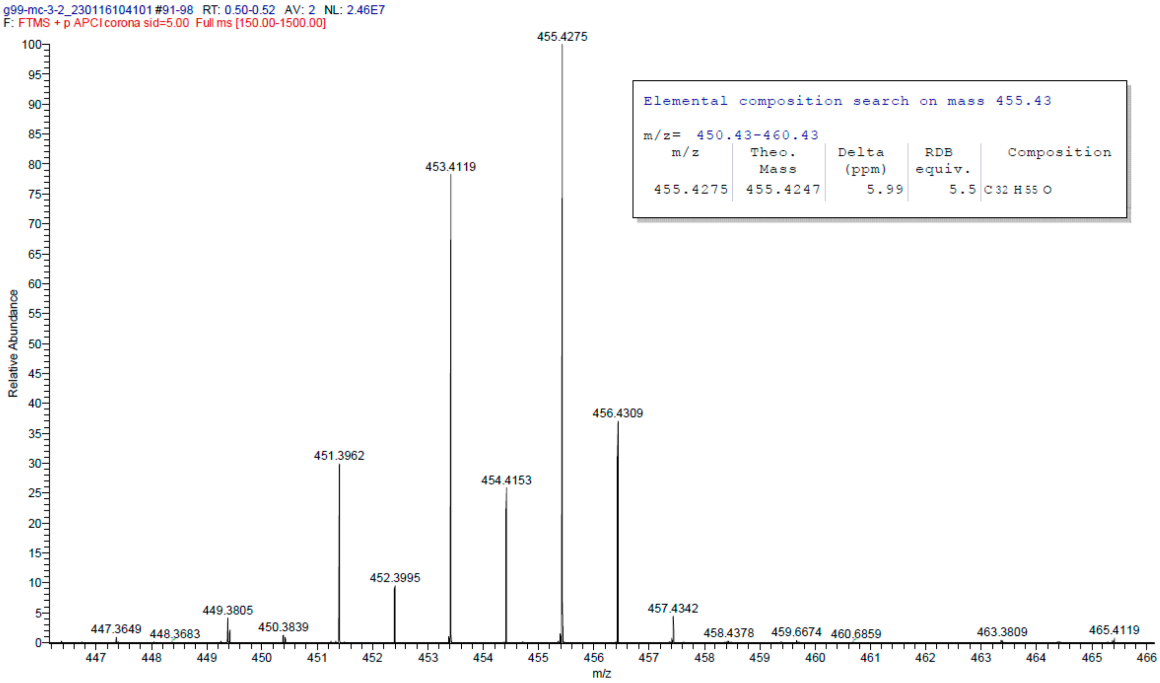

Figure S49. HRESIMS spectrum of compound **17**

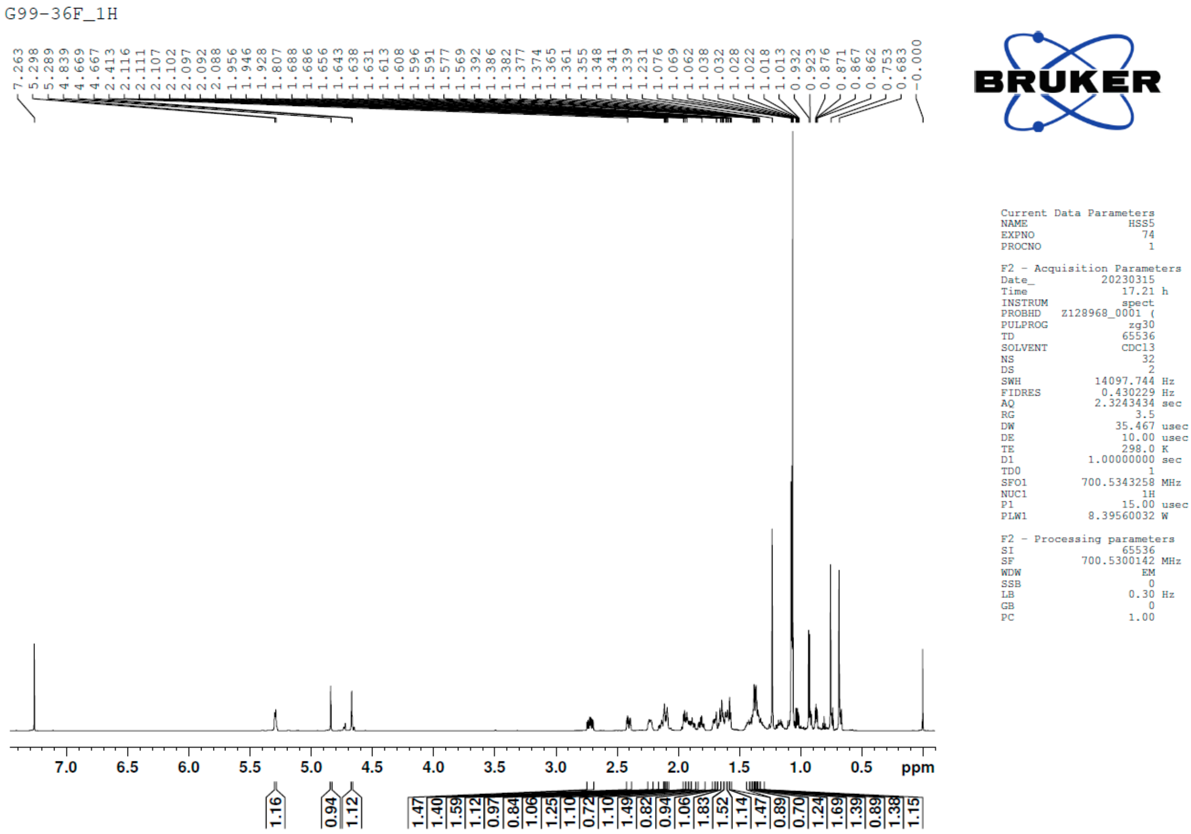

Figure S50. ^{1}H-NMR spectrum ($CDCl_3$, 700 MHz) of compound **18**

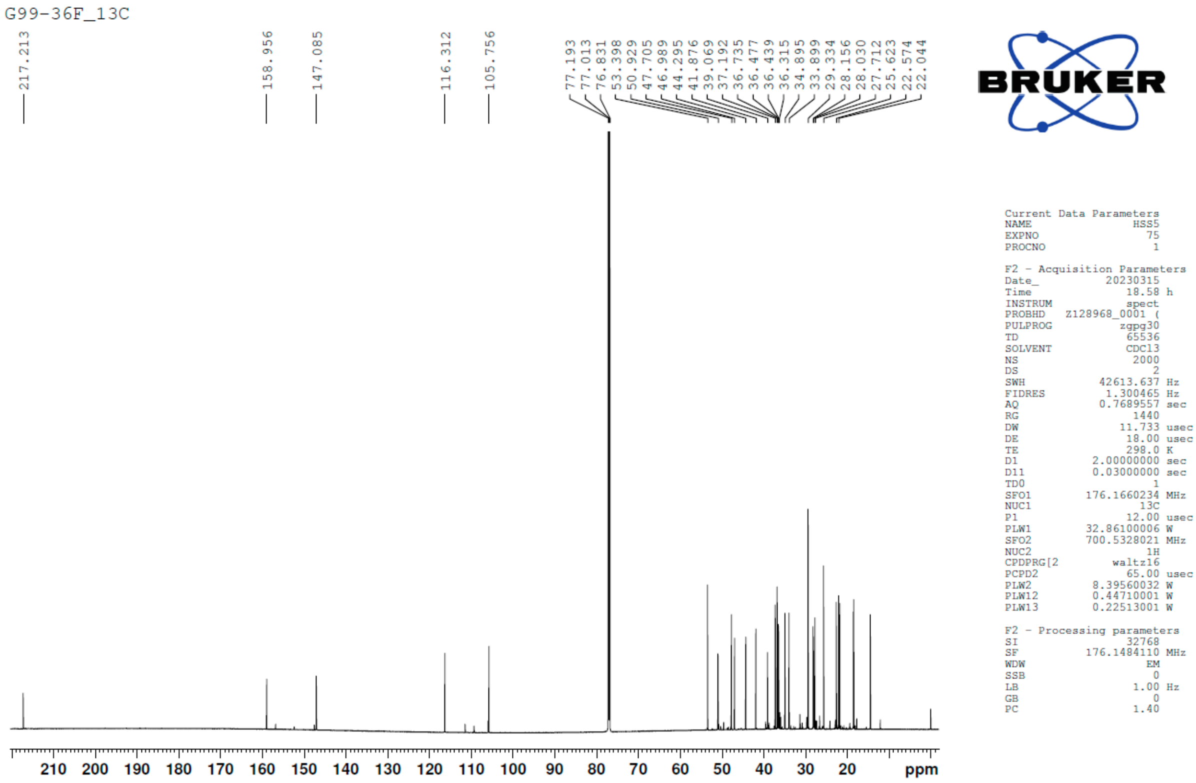

Figure S51. ^{13}C-NMR spectrum ($CDCl_3$, 175 MHz) of compound **18**

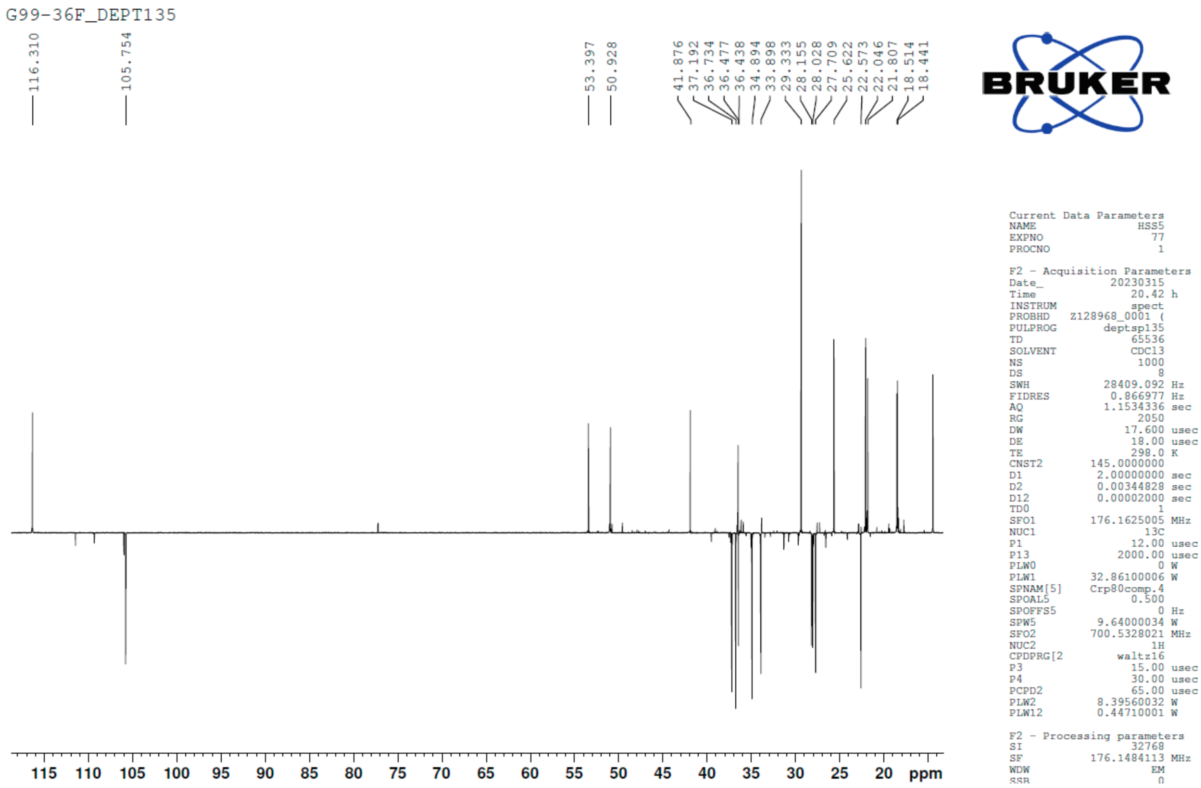

Figure S52. DEPT135 spectrum of compound **18**

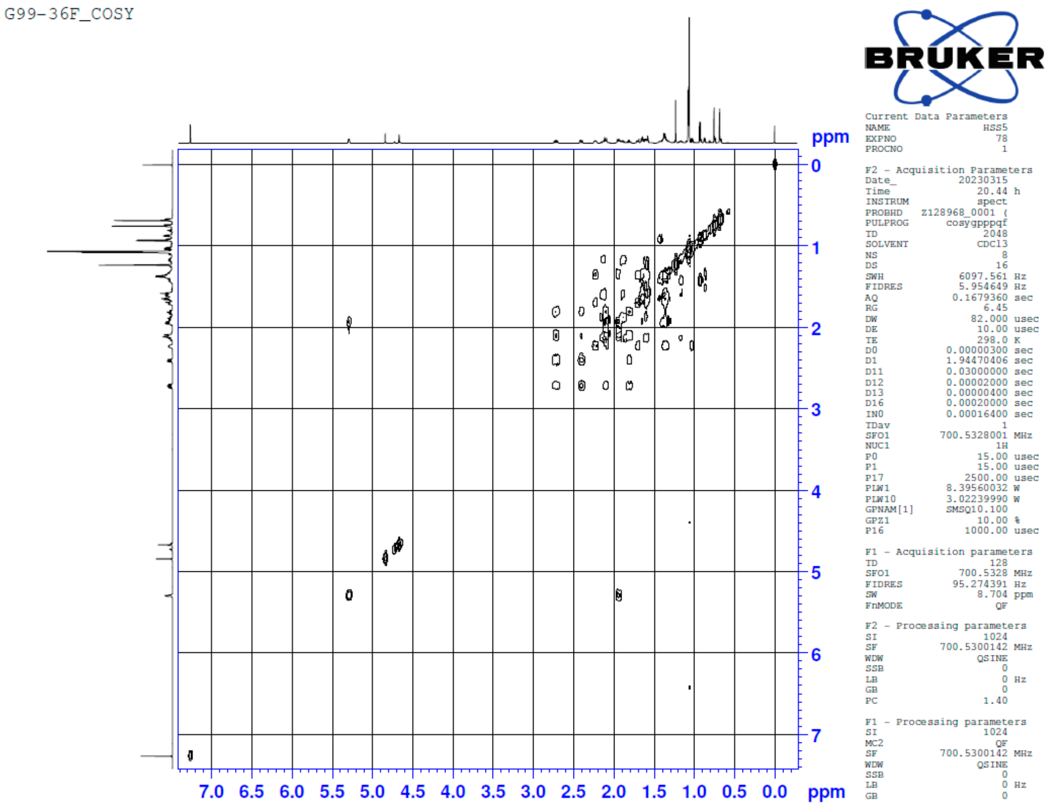

Figure S53. ^{1}H-^{1}H COSY spectrum of compound **18**

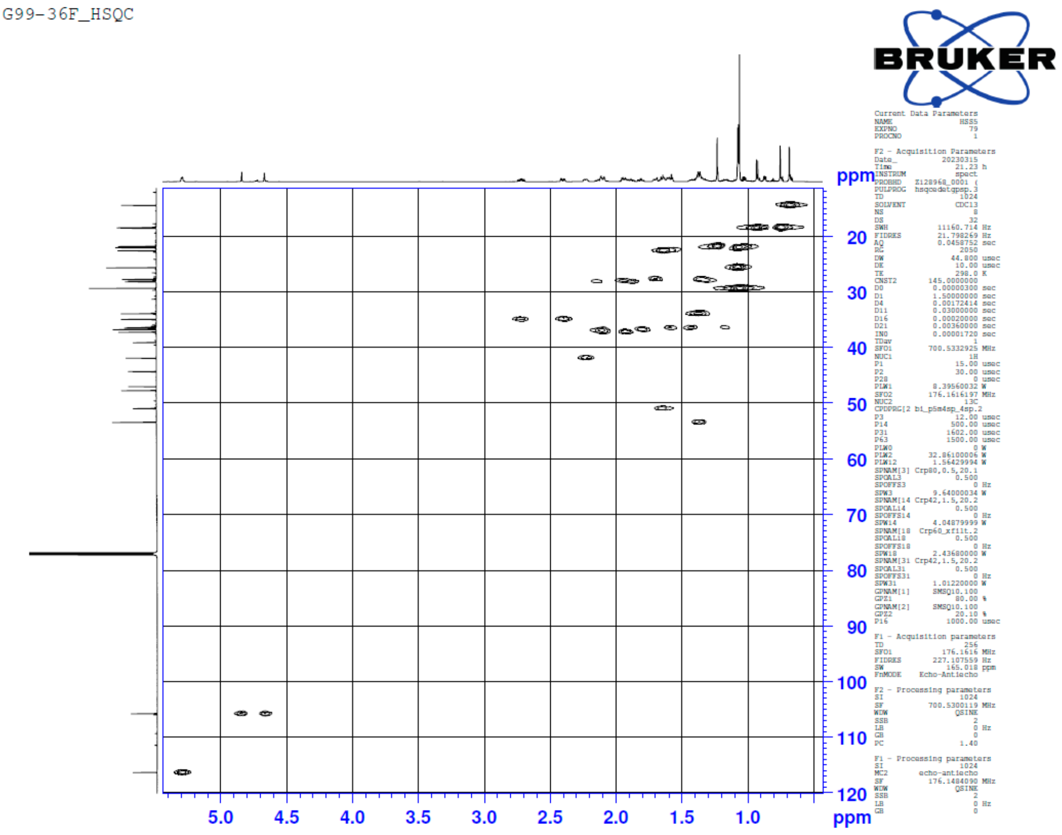

Figure S54. HSQC spectrum of compound **18**

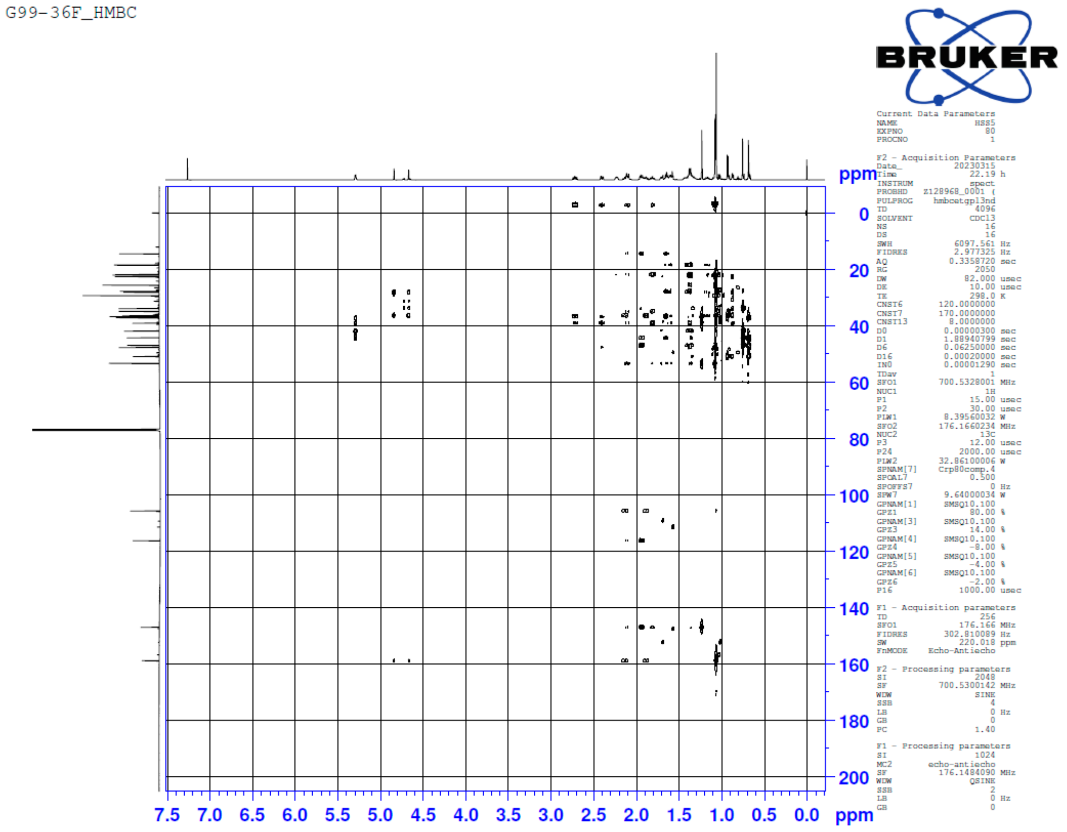

Figure S55. HMBC spectrum of compound **18**

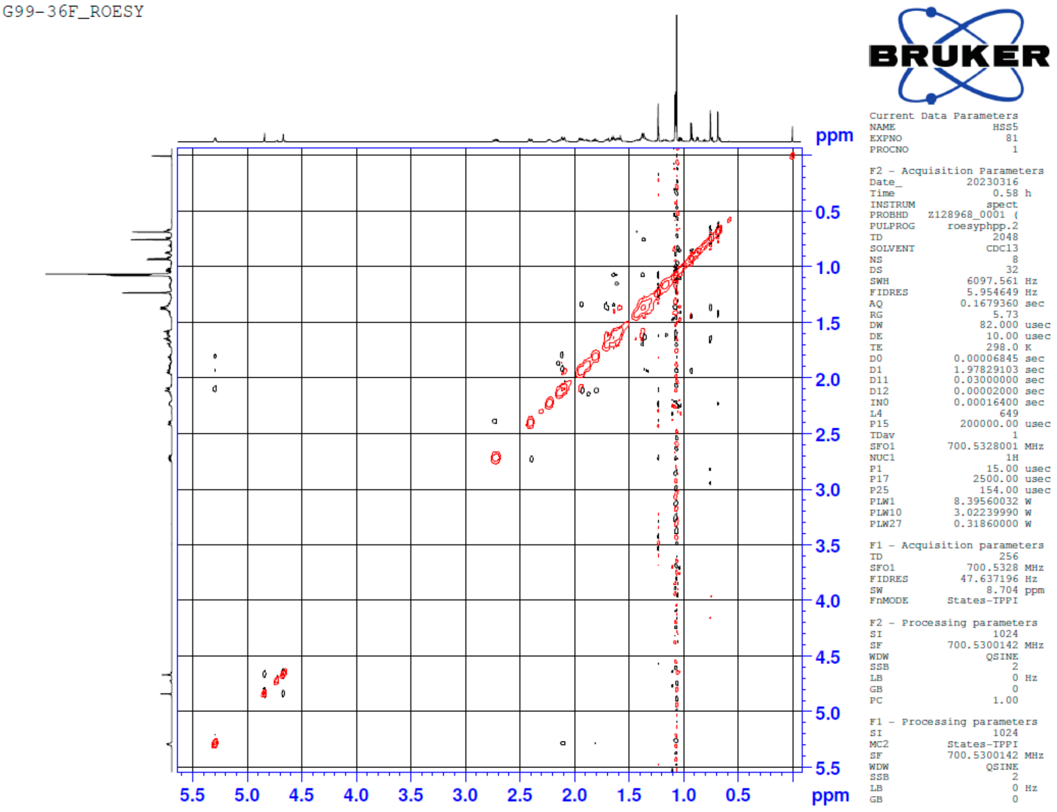

Figure S56. ROESY spectrum of compound **18**

Light Source	Na
Monitor wavelength	589 nm
D.I.T.	5 sec
No. of cycle	5
Cycle interval	0 sec
Temp. Monitor	Cell
Temp. Corr. Factor	None
Aperture(S)	8.0mm
Aperture(L)	Auto
Mode	Specific O.R.
Path Length	50 mm
Concentration	0.357143 w/v%
Water content of sample	0 %
Factor	1

		No.	Sample No.	Mode	Calc. Data	Meas. Data	Monitor(deg)	PMT Voltage[V]	Temperature(C)
1	*	1	G99-36_1-1	Specific O.R.	28.9408	0.0517	0.0527	303	23.27
2	*	2	G99-36_1-2	Specific O.R.	26.7008	0.0477	0.0487	304	23.27
3	*	3	G99-36_1-3	Specific O.R.	24.5728	0.0439	0.0449	303	23.27
4	*	4	G99-36_1-4	Specific O.R.	25.0768	0.0448	0.0458	304	23.27
5	*	5	G99-36_1-5	Specific O.R.	26.8128	0.0479	0.0489	304	23.27
6	*	6	Avg.		26.4208				
7		7	S.D		1.7178				
8		8	C.V		6.5018				

Figure S57. Specific optical rotation of compound **18**

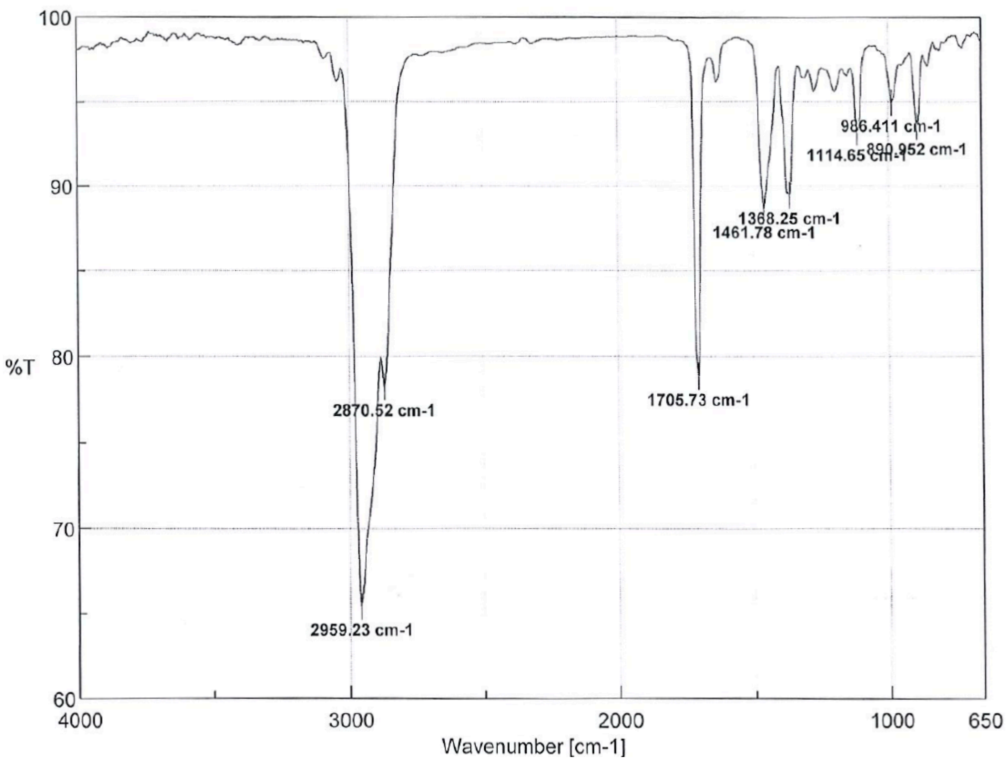

Figure S58. IR spectrum of compound **18**

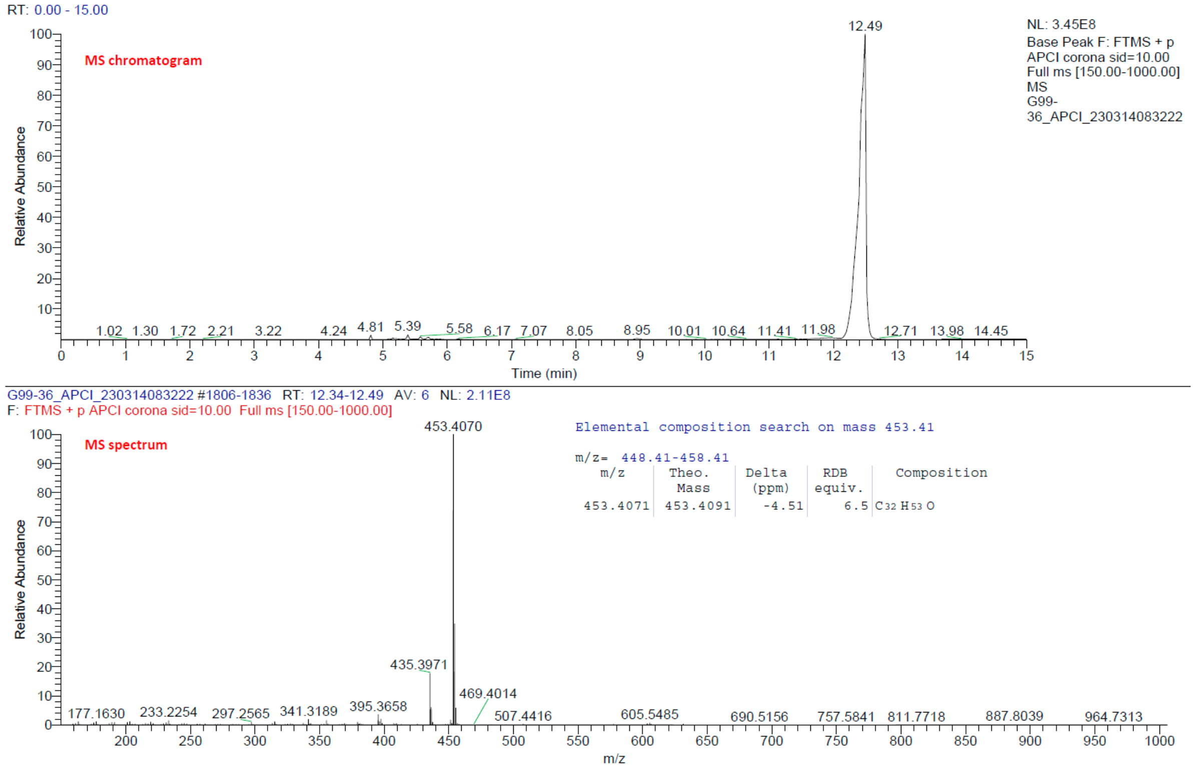

Figure S59. HRESIMS spectrum of compound **18**

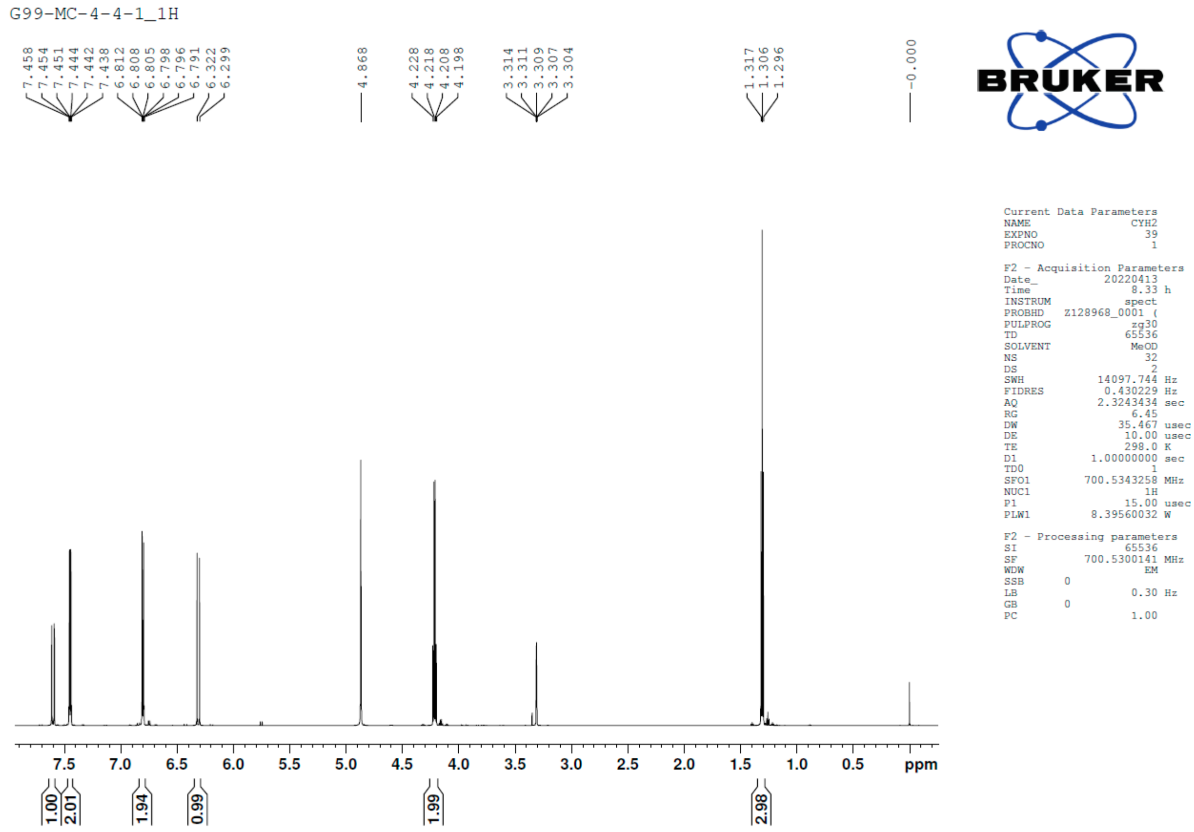

Figure S60. ^{1}H-NMR spectrum (CD_3OD, 700 MHz) of compound **19**

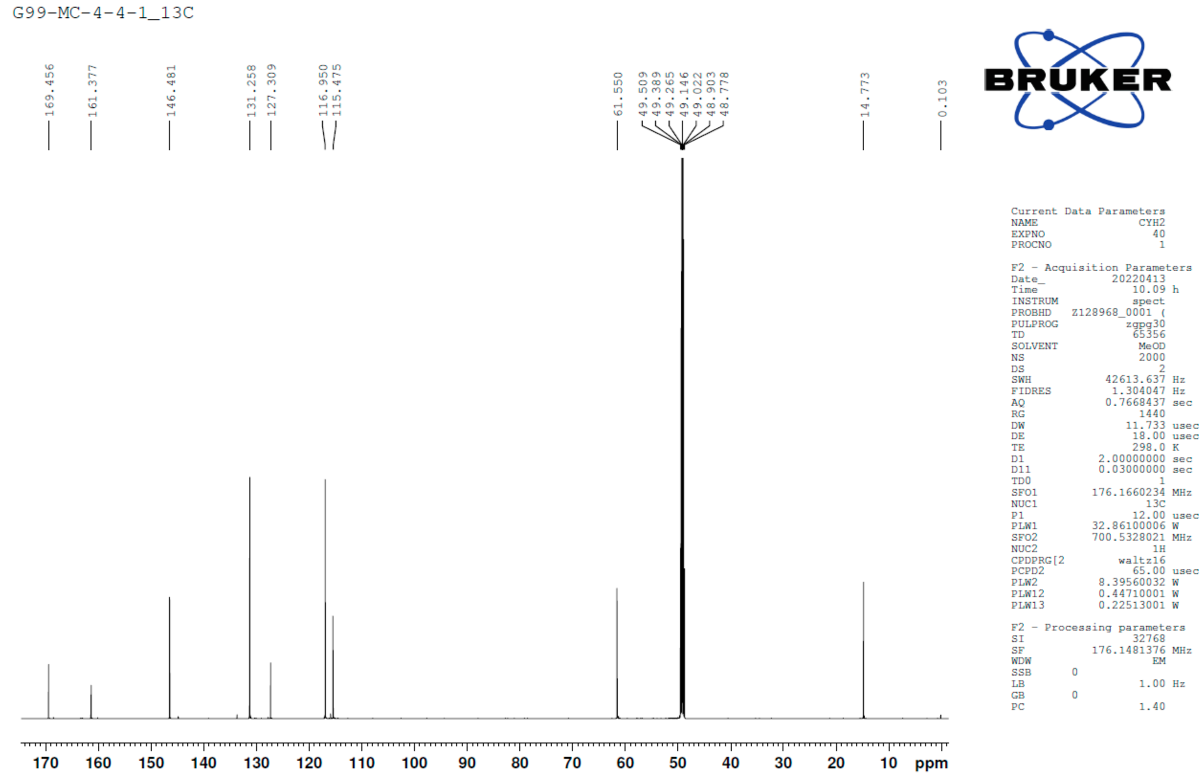

Figure S61. ^{13}C-NMR spectrum (CD_3OD, 175 MHz) of compound **19**
